# Machine learning-based models for screening of anemia and leukemia using features of complete blood count reports

**DOI:** 10.1038/s41598-025-21279-w

**Published:** 2025-09-29

**Authors:** Hafsa Amjad, Zamir Hussain, Mahnoor Hasan, Mahmood Ul Hassan

**Affiliations:** 1 Department of Sciences, School of Interdisciplinary Engineering and Sciences (SINES), National University of Sciences and Technology (NUST), Islamabad, 44000 Pakistan; 2 Division of Biostatistics, Institute of Environmental Medicine, Karolinska Institutet, Stockholm, Sweden

**Keywords:** Anemia, CBC reports, Clinical decision support, Leukemia, Machine learning, Cancer, Cancer screening

## Abstract

Complete blood count (CBC) report features are routinely used to screen a wide array of hematological disorders. However, the complexity of disease overlap increases the probability of neglecting the underlying patterns between these features, and the heterogeneity associated with the subjective assessment of CBC reports often lead to random clinical testing. Such disease prediction analyses can be enhanced by the incorporation of machine learning (ML) algorithms for efficient handling of CBC features. Hybrid synthetic data are generated based on the statistical distribution of features to overcome the constraint of small sample size (*N* = 287). To the extent of our knowledge, our study is the first to employ hybrid synthetic data for modeling hematological parameters. Six ML models i.e., decision tree, random forest, support vector machine, logistic regression, gradient boosting machine, and multilayer perceptron are tested for disease prediction. This research presents ML-based models for the screening of two common blood disorders – anemia and leukemia, using CBC report features. A ‘fingerprint’ of 14 out of 21 features based on both statistical and clinical relevance is selected for model development. Exceptional performance has been observed by the random forest algorithm with 98% accuracy and 97, 98, 99, and 2% macro-averages of precision, recall, specificity, and miss-rate respectively for all classes. However, external validation of the model reveal poor generalizability on a different demographic dataset, as the model obtained an accuracy of 74%. The proposed methodology may serve as an efficient support system for the screening of anemia and leukemia. However, extensive optimization with regards to its generalizability are warranted.

**Supplementary Information:**

The online version contains supplementary material available at 10.1038/s41598-025-21279-w.

## Introduction

The advent of Artificial Intelligence (AI) has immensely affected the approach to solving a wide range of data-related problems. With the ability to mimic human cognitive processes, AI and its branch of Machine Learning (ML) have undergone substantial developments in healthcare and medical research. ML works by finding the hidden underlying patterns and trends in large amounts of data, which assists in clinical decision-making^1–3^. The ‘learning’ and ‘self-correcting’ abilities of ML algorithms can also lead to the reduction of inevitable diagnostic and therapeutic errors^4–6^. Medical research has made several notable breakthroughs in the use of AI to diagnose and evaluate major diseases like cancer^7^, nervous system disorders^8,9^, and cardiovascular diseases^1^. However, there is a dire need to expand this knowledge to predict the complexities of disease overlap in hematological disorders as well.

Complete Blood Count (CBC) with a differential is a routinely performed baseline test, which provides comprehensive numerical estimates of blood cell counts, hemoglobin, hematocrit, and red blood cell indices^10^. It is used to assess a wide array of blood disorders including anemia, infections, inflammations, and blood cancers^10–13^. This study investigates the overlap between two of such disorders – anemia and leukemia. Anemia is a condition in which there is a shortage of red blood cells (RBCs) and hemoglobin, leading to a decreased capacity for carrying oxygen from the lungs to different parts of the body^14^. Anemia, on its own, is relatively a benign condition. However, it often results due to some serious underlying condition like leukemia, which is a type of blood cancer affecting all types of cells such as white blood cells (WBCs), RBCs, and platelets^15^. The differential diagnosis of leukemia is wide-ranging as it presents non-specific symptoms. To confirm its presence, it is important to rule out other blood-related disorders that can also disrupt the normal estimates of the blood cells in the body^15^. While evaluating a CBC report, healthcare professionals tend to focus only on the parameters outside the normal range. This increases the probability of neglecting the underlying patterns and correlations of one parameter with the other. This complexity of disease overlap, the expertise of healthcare professionals, and heterogeneity associated with the subjective assessment of a CBC report often lead to random clinical testing^16^. It is a common malpractice, which not only exhausts financial and clinical laboratory resources but also delays correct diagnosis and treatment to some extent.

Such disease prediction analyses can be enhanced by the incorporation of ML algorithms for the efficient handling and utilization of these hematological parameters^17^. Several studies have been conducted in recent years that have shown promising results in terms of the accuracy of ML models for predicting hematological malignancies using cell population data (CPD)^17,18^.

This research aims to minimize the practice of random clinical testing through an AI-driven clinical decision support system, which can efficiently detect two of the most common blood disorders – anemia and leukemia. A unique subset of CBC report features has been used to develop a ‘fingerprint’ of disease predictors. To the extent of our knowledge, it is worth mentioning that our study is the first one to employ hybrid synthetic data to overcome the constraints of small sample sizes that most recent studies encountered. To evaluate the performance of ML algorithms, stratified 5-fold cross-validation is performed, and metrics like accuracy, precision, recall, specificity, and miss-rate are used. External validation is also performed on the best-performing algorithm to assess the generalizability of the model. Such a smart system would lead to the timely detection of these two disorders and reduce the risk of patients being exposed to random clinical testing.

## Methods

**Fig. 1:**
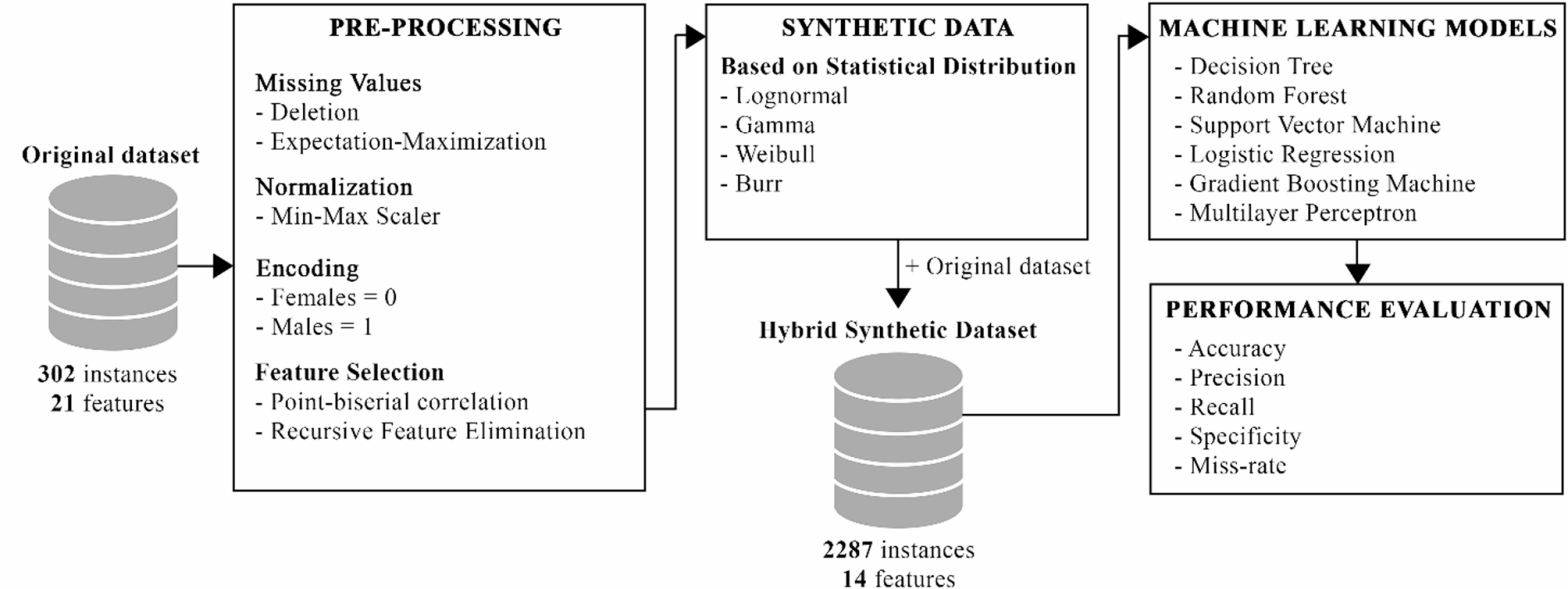
The overall workflow of the proposed methodology.

The overall methodology employed in this study is given in (Fig. 1).

### Data collection

The study subjects consisted of patients who visited different laboratories for CBC investigation in Islamabad and Rawalpindi, Pakistan. Data collection has been done from March to September, 2024. 302 random CBC reports of such patients are selected for this study to classify anemia and leukemia. Informed consent was obtained from the participants detailing their right to withdraw at any point and merits of anonymity and confidentiality (a copy of the consent form is provided in Supplementary Files). Ethical approval has been obtained from the review board of National University of Sciences and Technology under Application No. 2024-IRB-A-05/05 on 22/02/2024. Research has been performed in accordance with the Declaration of Helsinki.

The dataset consists of CBC reports of normal people and those suffering from anemia, leukemia, and both. 19 CBC report features and two demographic features (age and gender) are included. Four target classes – (1) Normal; (2) Anemia; (3) Leukemia; and (4) Combination, have been labeled in the data.

### Preprocessing

**Fig. 2:**
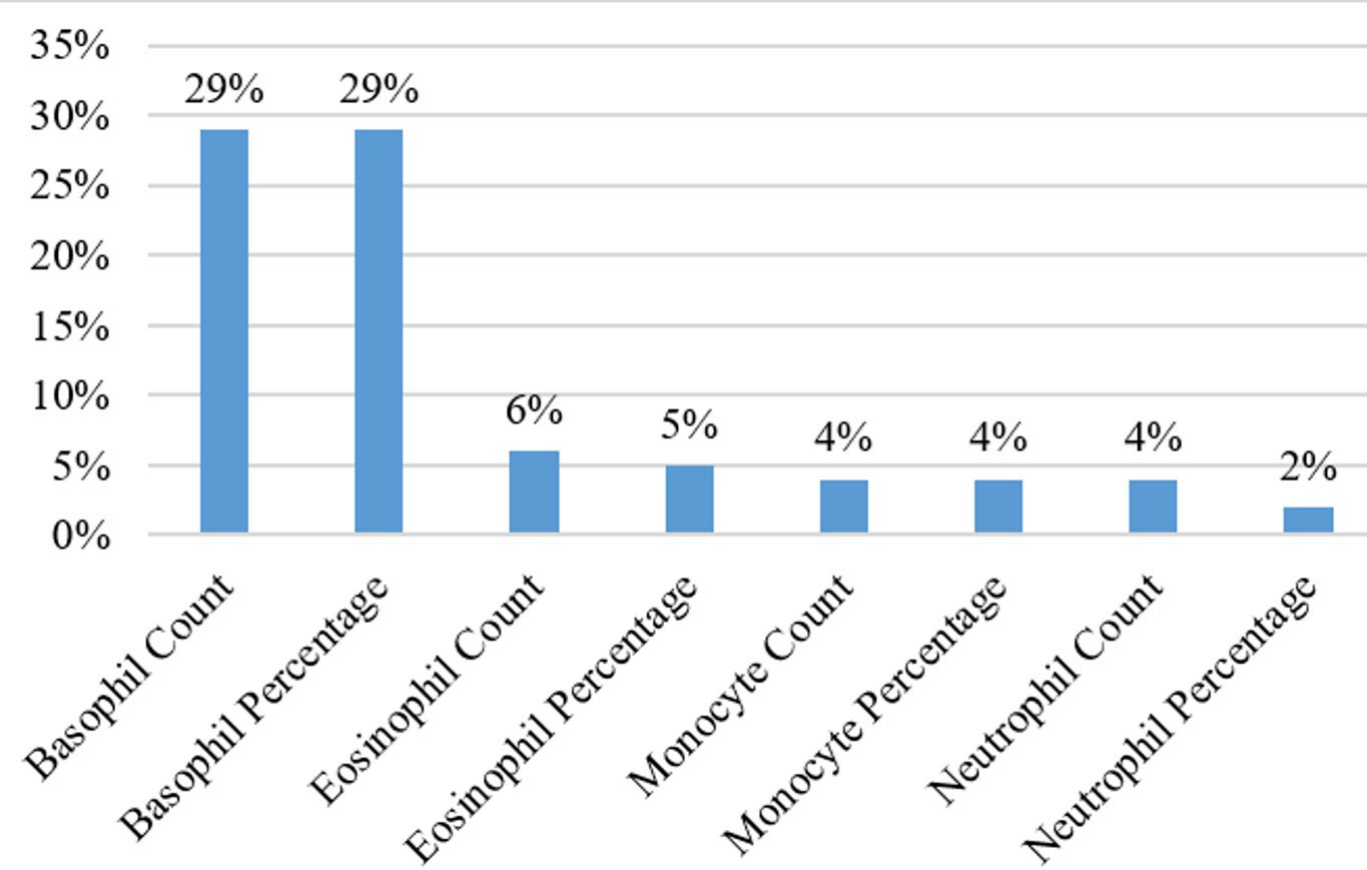
Percentage of missing values that were imputed in the data.

Several values in the dataset were missing. This issue is solved in two ways. First, all the instances and variables having more than 90% missing values are omitted. Consequently, 15 instances and one attribute, ‘Reticulocyte percentage’ are removed from the dataset. Second, Expectation Maximization (EM)^19^ is applied using IBM SPSS Statistics for Windows, version 20.0^20^, to predict the remaining missing values. EM has two modes of application: (1) Expectation mode: This step expects the missing values by using the parameters of the current probability distribution and finding the log-likelihood of the data; (2) Maximization mode: This step finds new parameters that maximize the log-likelihood found in the previous step. These two steps are iteratively applied until convergence to find the maximum log-likelihood and ultimately, the goodness-of-fit between the data and the model. Missing values are imputed for 8 features, which are described in Fig. 2 along with their corresponding percentages. After dealing with missing values, 287 cases with 18 hematological features and 2 demographic features (age and gender) are selected for further downstream analysis.

The values for hematological features of CBC reports have different units and ranges of measurement, therefore, the dataset needs to be normalized. For this, the Min-Max Scaler from the Scikit-learn library^21^ is used as a scaling approach to transform the range of values between 0 and 1. The dataset al.so contained one categorical demographic feature (gender), which is encoded in numerical form: 0 for female and 1 for male.

### Data description

The association of gender and three age groups (children, adults, and elderly) with the four target classes has revealed different results. Females in our dataset have a higher ratio in all target classes, as opposed to males. High prevalence rate of anemia, leukemia, and their combination can be seen in adult females belonging to the age group of 18–64 years. ‘Combination’ class is the most frequent in all three age groups. The demographic population distribution among the target classes is shown in Table 1.

**Table 1:** Demographic population distribution.

Age	Normal		Anemia		Leukemia		Combination		Total (%)
	Female (%)	Male (%)	Female (%)	Male (%)	Female (%)	Male (%)	Female (%)	Male (%)	
< 18 (Children)	4 (1.39)	2 (0.70)	0 (0)	0 (0)	0 (0)	1 (0.35)	19 (6.62)	32 (11.15)	58 (20.21)
18–64 (Adults)	25 (8.71)	13 (4.53)	16 (5.57)	1 (0.35)	15 (5.23)	11 (3.83)	74 (25.78)	47 (16.38)	202 (70.40)
65 + (Elderly)	5 (1.74)	1 (0.35)	0 (0)	0 (0)	1 (0.35)	0 (0)	17 (5.92)	3 (1.05)	27 (9.41)
Total	34 (11.85)	16 (5.57)	16 (5.57)	1 (0.35)	16 (5.57)	12 (4.18)	110 (38.32)	82 (30.31)	287 (100)

### Feature selection

To determine those features that have a significant impact on predicting anemia and leukemia, two feature selection approaches are utilized. First, point-biserial correlation using SPSS Statistics is applied to find features that are significantly correlated with the target variable. Point-biserial correlation is a filter-based approach, which is evaluated between − 1 and 1. The values closer to -1 indicate a strong negative correlation whereas the values closer to 1 indicate a strong positive correlation between two features. In this study, only the absolute correlation coefficient value is considered to rank the features (Table 2).

**Table 2:** CBC report features based on point-biserial correlation.

Features	Abbreviations	Absolute Correlation
Hemoglobin	HB	0.696
Hematocrit	HCT	0.686
Red blood cells	RBC	0.627
Monocyte percentage	MONO%	0.328
Platelet count	PLT	0.267
Eosinophil percentage	EO%	0.246
Monocyte count	MONO	0.245
Neutrophil percentage	NEUT%	0.237
White blood cells	WBC	0.222
Neutrophil count	NEUT	0.192
Lymphocyte count	LYM	0.175
Basophil count	BASO	0.166
Eosinophil count	EO	0.166
Basophil percentage	BASO%	0.142
Gender	Gender	0.123
Mean corpuscular hemoglobin concentration	MCHC	0.096
Lymphocyte percentage	LYM%	0.091
Age	Age	0.089
Mean corpuscular volume	MCV	0.043
Mean corpuscular hemoglobin	MCH	0.016

Second, Recursive Feature Elimination (RFE) is applied with three tree-based machine learning algorithms i.e., Decision Tree (DT), Random Forest (RF), and Gradient Boosting Machine (GBM) using Sci-kit learn library. RFE is a wrapper approach, in which a ML model is trained several times on different subsets of features. It starts with the complete set of features and on each iteration, it eliminates one feature. This approach gives the optimal number of features with which the model performs the best. Optimal features are selected based on the feature importance scores calculated by these tree-based algorithms.

Comparative analysis and assessment of the feature selection results is achieved by performing two common set operations i.e., intersection and union, on the sets of features obtained from point-biserial correlation and RFE. Evaluation of the mentioned set operations is performed by analyzing the accuracy, recall (diagnostic sensitivity), and false negative rate (diagnostic miss-rate) of the ML models. This results in a final subset of statistically relevant CBC report features, which are then validated by specialized healthcare professionals to add clinical relevance.

### Hybrid synthetic data generation

The dataset used in this study is small in size with only 287 instances. It is difficult to obtain labeled and annotated medical data due to privacy and ethical concerns. Therefore, synthetic data are generated in this study to improve the resilience and flexibility of the models^22,23^. Synthetic data are generated based on the statistical distributions followed by the selected CBC features for each target class using EasyFit 5.6 Professional^24^. Lognormal, gamma, Weibull, and burr distributions are selected to model the continuous and non-negative blood parameters based on literature support^25–27^. The details of these probability distributions are given in Supplementary Files (Figures S1-S4 in SDG.pdf). The validation of the goodness-of-fit of these distributions is achieved by Kolmogorov-Smirnov and Anderson Darling tests at an alpha level of 0.05. If the calculated statistical quantity of each of these tests is smaller than the critical value for that test, it indicates that the applied distribution matches the sample data for that particular CBC report feature. Detailed results are provided in Supplementary Files (Figures S5-S8 and Tables S1-S4 in SDG.pdf). Using the best-fitted distributions, new data points are then generated using a random number generator algorithm in EasyFit software. These new synthetic data points retain the distributional properties of the original data. For each target class, 500 instances are generated resulting in a total of 2000 synthetic instances. The synthetic data for each target class are then combined with the original data points to generate a ‘hybrid’ synthetic dataset consisting of 2287 instances. The class distribution of all datasets is given in Table 3.

**Table 3:** Distribution of target classes in original dataset, synthetic dataset, and hybrid synthetic dataset.

Target classes	Original dataset (*n* = 287)	Synthetic dataset (*n* = 2000)	Hybrid dataset (*n* = 2287)
Normal	50	500	550
Anemia	17	500	517
Leukemia	28	500	528
Combination	192	500	692

### Model selection

In this study, six machine learning models are applied – DT, RF, Support Vector Machine (SVM), Logistic Regression (LR), GBM, and Multi-layer Perceptron (MLP). These models are used from Scikit-learn library^21^ using Python version 3.10.12 as the programming language. Grid search is performed for hyperparameter tuning of each model, which primarily returned default hyperparameters as the most optimal configuration for the models (Table 4).

**Table 4:** Parameter grids and selected optimal hyperparameters obtained from grid search.

Algorithm	Parameter Grid	Output
DT	param_grid = {‘criterion’: [‘gini’, ‘entropy’], ‘max_depth’: [None, 5, 10, 20], ‘min_samples_leaf’: [1, 2, 4], ‘min_samples_split’: [2, 5, 10]}	{‘criterion’: ‘gini’, ‘max_depth’: 10, ‘min_samples_leaf’: 1, ‘min_samples_split’: 2}
RF	param_grid = {‘n_estimators’: [50, 100, 200], ‘criterion’: [‘gini’, ‘entropy’], ‘max_depth’: [None, 5, 10], ‘min_samples_leaf’: [1, 2], ‘min_samples_split’: [2, 5]}	{‘n_estimators’: 100, ‘criterion’: ‘gini’, ‘max_depth’: ‘None’, ‘min_samples_leaf’: 1, ‘min_samples_split’: 2}
GBM	param_grid = {‘n_estimators’: [50, 100, 200], ‘criterion’: [‘friedman_mse’, ‘squared_error’], ‘learning_rate’: [0.01, 0.1, 0.2], ‘max_depth’: [3, 5], ‘min_samples_leaf’: [1, 2], ‘min_samples_split’: [2, 5]}	{‘n_estimators’: 100, ‘criterion’: ‘friedman_mse’, ‘learning_rate’: 0.01, ‘max_depth’: 3, ‘min_samples_leaf’: 1, ‘min_samples_split’: 2}
SVM	param_grid = {‘C’: [1, 10, 20, 30], ‘kernel’: [‘rbf’, ‘poly’], ‘gamma’: [‘scale’, ‘auto’]}	{‘C’: 30, ‘kernel’: ‘rbf’, ‘gamma’: ‘scale’}
LR	param_grid = {‘C’: [0.01, 0.1, 1, 10], ‘penalty’: ‘l1’, ‘l2’, ‘elasticnet’, ‘solver’: [‘lbfgs’, ‘liblinear’]}	{‘C’: 0.01, ‘penalty’: ‘l2’, ‘solver’: ‘lbfgs’}
MLP	param_grid = {‘hidden_layer_sizes’: [(200, 100, 50), (100, 50, 10)], ‘activation’: [‘relu’, ‘tanh’], ‘alpha’: [0.0001, 0.001, 0.01] ‘max_iter’: [200, 500, 1000]}	{‘hidden_layer_sizes’: (100, 50, 10), ‘activation’: ‘relu’, ‘alpha’: 0.01 ‘max_iter’: 1000}

A few of the default hyperparameters were changed as follows:

- For DT, maximum depth is changed from default of **None** to **10**.
- For SVM, value of C (penalty on misclassification) is changed from default of **1.0** to **30**. Between the multiclass kernels **rbf** and **poly**, grid search returned **rbf** as the best hyperparameter.
- For LR, value of C (strength of regularization) is changed from default of **1.0** to **0.01.**
- For MLP, the architecture consists of three hidden layers with 100, 50, and 10 nodes respectively, with Rectified Linear Unit (ReLU) as the activation function. The maximum number of iterations is set to 1000. The value of alpha is changed from default of **0.0001** to **0.01**.

For all models, random seed of 42 is used for training. This resulted in the best performance for all models (See Tables S1-S3 in Supplementary File “Seed-Trials.pdf).

### Performance evaluation

The performance evaluation of the ML models is done using stratified 5-fold cross-validation. This approach is mainly used when the dataset is small and class-imbalanced. The dataset is split into k-folds (k = 5), ensuring that each fold has the same proportion of observations of the target class. The process is repeated 5 times with a different fold as the testing set and the remaining k-1 folds for training the ML models. The performance results are then averaged over all the repetitions. 95% Confidence Intervals (CI) of the metrics are also reported.

In this study, five standard performance metrics namely accuracy, precision, recall, specificity, and false negative rate/miss-rate are used for the assessment analysis of different classification techniques. Receiver Operating Characteristic (ROC) curve and Area under the ROC curve (AUC) are also visualized for each of the 4 target classes. As ROC and AUC are natively binary metrics, the curves are plotted using the One-vs-Rest approach to reduce the multiclass problem to a binary one. Paired t-test are also applied at a significance level of 0.05 between the performance metrics of models to determine statistically significant differences.

### External validation

For external validation of the top-performing model, a secondary dataset of 300 CBC reports of patients in Iraq is used^28^. This is an unlabeled dataset, publically available on Mendeley Data. It consists of 28 variables including gender and CBC report features. After preprocessing and removal of missing values and outliers, 30 instances are removed, resulting in an external validation dataset of 270 instances. Specialized healthcare professionals have labeled the dataset for the 4 target classes of Normal, Anemia, Leukemia, and Combination based on ranges of different hematological parameters. Distribution of these target classes is defined in Table 5. This provides the actual or true labels for the dataset. The unlabeled dataset is used for external validation of the final selected algorithm. This generates predicted labels for the dataset, which are then compared with the actual labels to determine the recall or true positive rate (TPR) of the final model.

**Table 5:** Distribution of target classes in external validation dataset.

Target classes	Frequency
Normal	54
Anemia	136
Leukemia	18
Combination	62

## Results

### Feature selection

Evaluation of the feature selection process is done in several ways. First, different thresholds of the point-biserial correlation coefficients i.e., 0.1, 0.2, and 0.3, are specified. All features below the specified correlation threshold are omitted from the dataset. These thresholds are then tested by using three tree-based ML algorithms – DT, RF, and GBM. The results show that the highest accuracy and recall rate are achieved by the RF algorithm for all the tested thresholds. Features having a correlation coefficient equal to or greater than 0.1 performs the best with an accuracy of 88% and a recall rate of 73%. In contrast, features having correlation coefficients equal to or greater than 0.2 and 0.3 result in lower accuracy and recall rates. Therefore, feature selection with a threshold of 0.1 is considered for further downstream analysis. Such feature selection eliminates 5 features from the total of 20 as shown in Table 6.

**Table 6:** Total predictor features and the results of the best-performing ML model for different coefficient thresholds.

Used features	Total predictor features	Model	Accuracy	Recall
All variables	20	RF	0.87	0.66
≥ 0.1	15	RF	0.88	0.73
≥ 0.2	9	RF	0.82	0.58
≥ 0.3	4	RF	0.80	0.51

Secondly, RFE with DT, RF, and GBM as an estimator result in the best performance with 15, 13, and 7 predictor features with an accuracy of 89%, 93%, and 91% respectively. After performing point-biserial correlation analysis and RFE, four sets of features are obtained. The performance results of intersection and union operations on the set of features obtained from the point-biserial correlation method with the three sets of features obtained from RFE are shown in Table 7.

**Table 7:** Performance results of set operations on the four feature sets obtained from point-biserial correlation and RFE.

Set operation	Metrics	Feature selection approach		
		Point-biserial + RFE(DT)	Point-biserial + RFE(RF)	Point-biserial + RFE(GBM)
Intersection	n	11	12	7
	Accuracy	0.87	0.88	0.90
	Recall	0.76	0.70	0.77
	Miss-rate	0.24	0.30	0.23
Union	n	19	16	15
	Accuracy	0.84	0.89	0.87
	Recall	0.69	0.71	0.73
	Miss-rate	0.30	0.29	0.28

On comparing these results, it is evident that the performance of ML models is either unchanged or enhanced when those features are considered that are commonly suggested by both the feature selection methods (intersection sets). To obtain a definitive and single set of predictor features that can efficiently classify the four target classes, the three intersection sets are further evaluated. The results show that the intersection set containing seven features performs the best with higher accuracy and recall rates and lower miss-rates (Table 8).

**Table 8:** Performance results of set operations on the three intersection sets of point-biserial and RFE.

Selected sets of features	Set operation	Metrics	Models		
			DT	RF	GBM
Intersection sets of point-biserial with RFE (DT), RFE (RF), and RFE (GBM)	Intersection	n	7		
		Accuracy	0.88	0.89	0.90
		Recall	0.73	0.75	0.77
		Miss-rate	0.26	0.25	0.23
	Union	n	12		
		Accuracy	0.87	0.88	0.88
		Recall	0.75	0.70	0.73
		Miss-rate	0.24	0.30	0.27

**Fig. 3:**
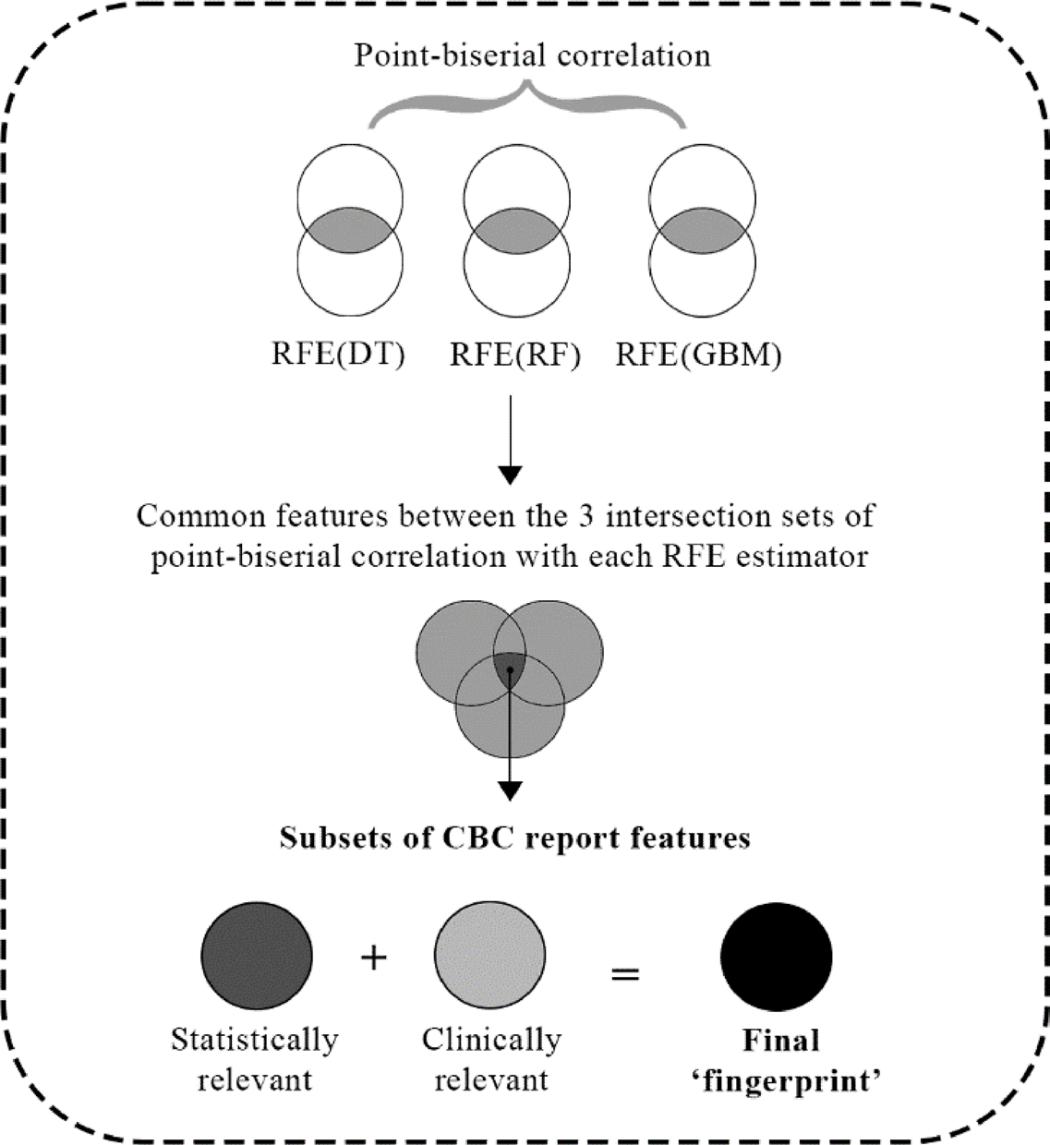
Selection of the final fingerprint of the CBC features.

The final pool of selected features for the predictive modeling of anemia and leukemia is obtained from the combination of statistically and clinically significant features (Table 9; Fig. 3). Such an approach to feature selection results in a total of 14 features that are used to train the ML models.

**Table 9:** Final pool of selected features from the combination of statistically and biologically significant features. The cross (✖) marks indicate that the features were not statistically significant/biologically significant/selected in the final pool of features. The check (✔) marks indicate that the features were statistically significant/biologically significant/selected in the final pool of features.

All features	Statistically significant features	Biologically significant features	Final pool of features
Gender	✖	✖	✖
Age	✖	✖	✖
WBC	✖	✔	✔
RBC	✖	✔	✔
HB	✔	✔	✔
HCT	✔	✔	✔
MCV	✖	✔	✔
MCH	✖	✔	✔
MCHC	✖	✔	✔
PLT	✔	✖	✔
NEUT	✔	✔	✔
LYM	✖	✔	✔
BASO	✔	✖	✔
EO	✖	✖	✖
MONO	✔	✖	✔
NEUT%	✖	✖	✖
LYM%	✖	✔	✔
BASO%	✖	✖	✖
EO%	✖	✖	✖
MONO%	✔	✖	✔

### Model selection

For the original dataset with 287 instances and 14 selected features, the RF algorithm is observed to be the best performing among all ML models with an accuracy of 98%. The highest recall rate is achieved by the ‘combination’ class while the ‘anemia’ and ‘leukemia’ classes show relatively poor recall rates with increased percentages of false negatives (miss-rates).

In case of the hybrid synthetic data, results have significantly improved due to increased data size and minimal class imbalance. RF and GBM have the best performance among the ML models with an accuracy of 98% and 97% respectively. Concerning other performance metrics such as precision, recall, specificity, and miss-rate, the majority of algorithms show promising results (Table 10).

**Table 10:** Performance metrics of the six ML models trained on the hybrid synthetic dataset with selected features.

Model	Accuracy	Classes	Precision	Recall	Specificity	Miss-rate
DT	0.947 ± 0.008	Normal	0.913 ± 0.021	0.967 ± 0.018	0.971 ± 0.008	0.033 ± 0.018
		Anemia	0.957 ± 0.025	0.959 ± 0.022	0.987 ± 0.008	0.041 ± 0.022
		Leukemia	0.945 ± 0.014	0.899 ± 0.040	0.984 ± 0.005	0.101 ± 0.040
		Combination	0.971 ± 0.011	0.957 ± 0.016	0.987 ± 0.005	0.043 ± 0.016
RF	0.980 ± 0.007	Normal	0.975 ± 0.015	0.978 ± 0.014	0.992 ± 0.005	0.022 ± 0.014
		Anemia	0.990 ± 0.008	0.986 ± 0.010	0.997 ± 0.002	0.014 ± 0.010
		Leukemia	0.964 ± 0.019	0.968 ± 0.014	0.989 ± 0.006	0.032 ± 0.014
		Combination	0.988 ± 0.006	0.986 ± 0.004	0.995 ± 0.002	0.014 ± 0.004
GBM	0.971 ± 0.008	Normal	0.938 ± 0.020	0.984 ± 0.007	0.979 ± 0.007	0.016 ± 0.007
		Anemia	0.975 ± 0.012	0.988 ± 0.007	0.993 ± 0.004	0.012 ± 0.007
		Leukemia	0.972 ± 0.010	0.937 ± 0.023	0.992 ± 0.003	0.063 ± 0.023
		Combination	0.994 ± 0.003	0.973 ± 0.008	0.997 ± 0.001	0.027 ± 0.008
SVM	0.879 ± 0.021	Normal	0.830 ± 0.026	0.891 ± 0.025	0.942 ± 0.010	0.109 ± 0.025
		Anemia	0.808 ± 0.047	0.983 ± 0.013	0.931 ± 0.020	0.017 ± 0.013
		Leukemia	0.904 ± 0.031	0.746 ± 0.036	0.976 ± 0.008	0.254 ± 0.036
		Combination	0.983 ± 0.009	0.893 ± 0.036	0.993 ± 0.004	0.107 ± 0.036
LR	0.816 ± 0.024	Normal	0.759 ± 0.043	0.827 ± 0.031	0.915 ± 0.022	0.173 ± 0.031
		Anemia	0.727 ± 0.040	0.840 ± 0.037	0.907 ± 0.016	0.160 ± 0.037
		Leukemia	0.836 ± 0.044	0.686 ± 0.012	0.959 ± 0.013	0.314 ± 0.012
		Combination	0.942 ± 0.015	0.889 ± 0.045	0.976 ± 0.006	0.111 ± 0.045
MLP	0.879 ± 0.018	Normal	0.854 ± 0.051	0.844 ± 0.086	0.952 ± 0.023	0.156 ± 0.086
		Anemia	0.801 ± 0.069	0.897 ± 0.069	0.931 ± 0.035	0.103 ± 0.069
		Leukemia	0.898 ± 0.030	0.816 ± 0.050	0.972 ± 0.010	0.184 ± 0.050
		Combination	0.970 ± 0.010	0.941 ± 0.021	0.987 ± 0.004	0.059 ± 0.021

**Fig. 4:**
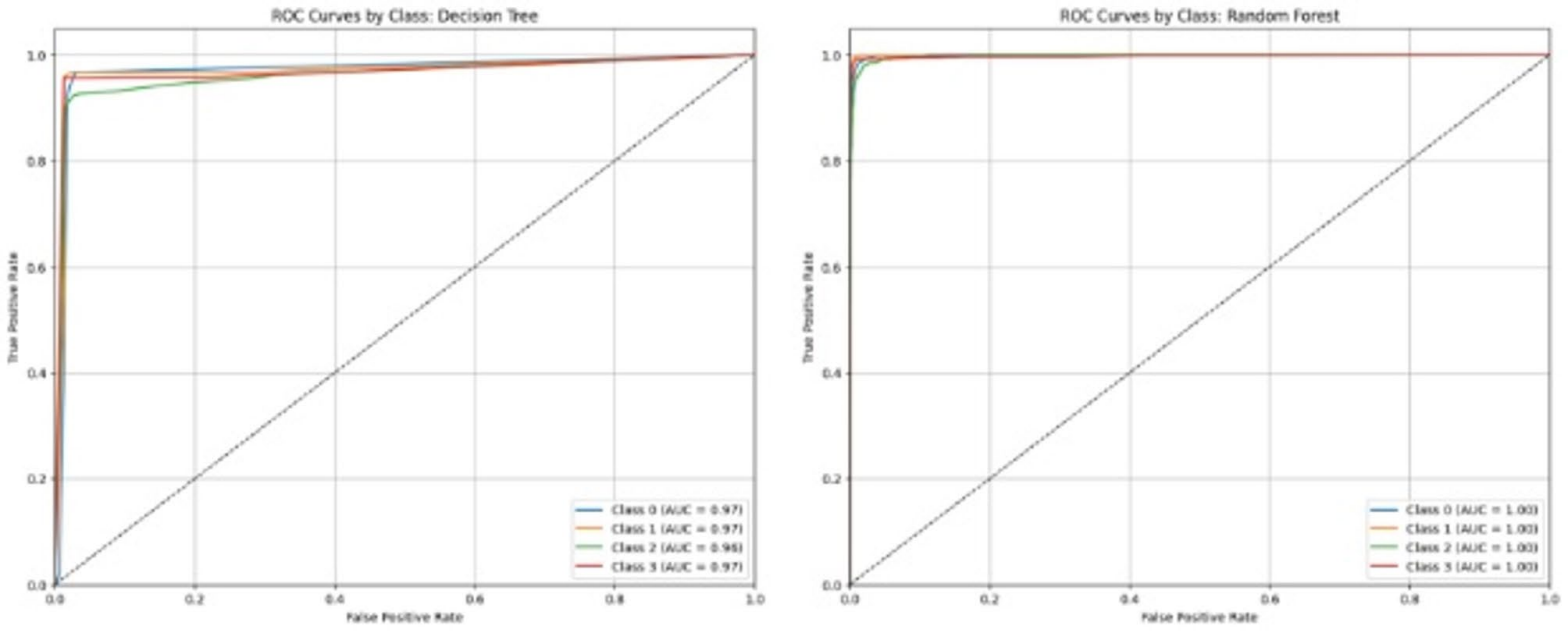
Receiver operating characteristic (ROC) curves and area under ROC curve (AUC) of the 4 target classes with decision tree (left) and random forest (right).

**Fig. 5:**
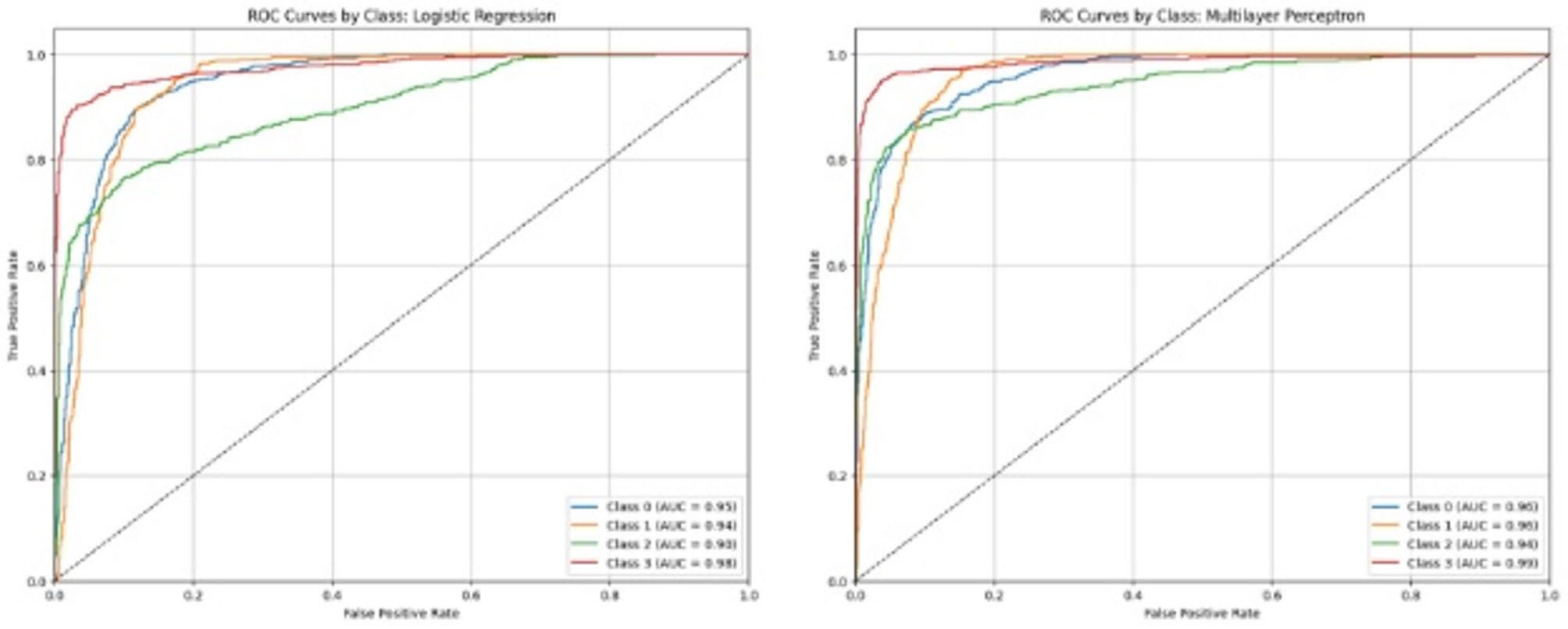
Receiver operating characteristic (ROC) curves and area under ROC curve (AUC) of the 4 target classes with logistic regression (left) and multilayer perceptron (right).

**Fig. 6:**
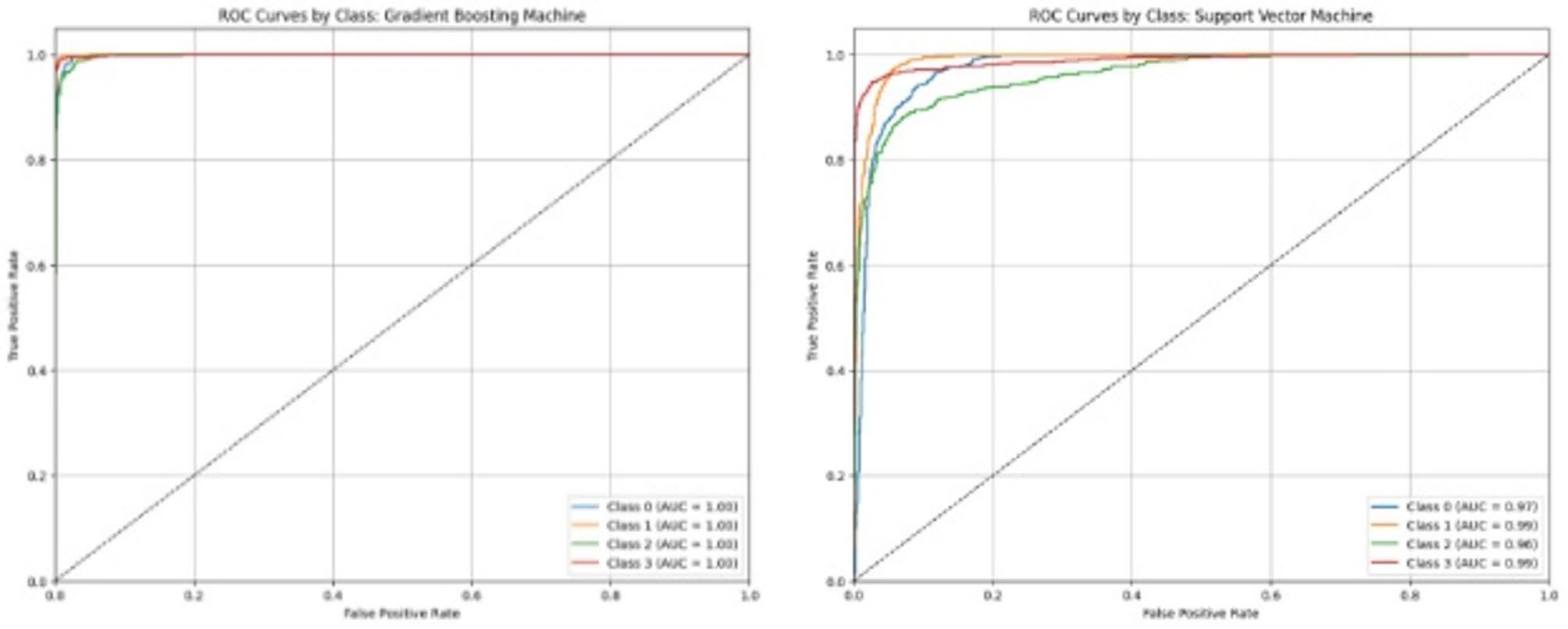
Receiver operating characteristic (ROC) curves and area under ROC curve (AUC) of the 4 target classes with gradient boosting machine (left) and support vector machine (right).

The ROC curve and AUC of all four target classes observed in each of the 6 algorithms are shown in Figs. 4, 5 and 6. These also describe the same trends of performance as Table 9, where both RF and GBM are the top-performing models with AUCs equal to 1 for each class. From Table 9, it is clear that RF (accuracy = 98%) is slightly better than GBM (accuracy = 97%). A paired t-test applied between the performance of these two models also show statistically significant difference at the 0.05 level (Table 11). Therefore, Random Forest is chosen as the top-performing model in the study.

**Table 11:** Results of paired t-tests applied between the performance metrics of Random Forest and Gradient Boosting Machine. Asterisk (*) indicate statistically significant results.

Performance metric	t-statistic	*p*-value
Accuracy	20.125	0.000*
Macro average of precision	23.401	0.000*
Macro average of recall	3.045	0.038*
Macro average of specificity	7.253	0.001*
Macro average of miss-rate	-2.901	0.044*

### External validation

The RF model is used to predict the labels on the external validation dataset. These predicted labels are compared with the labels assigned by clinicians. The confusion matrix of the RF model applied on external validation dataset is shown in Table 12 and performance metrics are described in Table 13.

**Table 12:** Confusion matrix showing the predictions of the random forest model on the external validation dataset.

Class labels		Predicted			
		Normal	Anemia	Leukemia	Combination
Actual	Normal	42	0	12	0
	Anemia	1	85	14	36
	Leukemia	2	0	16	0
	Combination	0	0	3	59

**Table 13:** Performance metrics of the final RF model on external validation dataset.

Accuracy	Classes	Precision	Recall	Miss-rate	F1 score
0.748	Normal	0.933	0.778	0.222	0.849
	Anemia	1.000	0.625	0.375	0.769
	Leukemia	0.355	0.889	0.111	0.507
	Combination	0.621	0.952	0.048	0.752

The final RF model has an accuracy of 75% on the external validation dataset, which is a significant drop as compared to the accuracy obtained with the hybrid synthetic data. While the recall (TPR) for the classes Leukemia and Combination are high with values of 89% and 95%, respectively, the recall of ‘Normal’ and ‘Anemia’ classes are quite low at 78% and 63%, respectively. The confusion matrix in Table 12 shows that 12 true Normal instances are misclassified as Leukemic, and a significant proportion of true Anemic cases are misclassified as Leukemic and the Combination class. This indicates that the model is over-fit for the Leukemia and Combination classes, which has decreased its overall performance.

## Discussion

AI-driven predictive modeling has widespread applications in both medical and health research^29,30^. The most common applications of ML in clinical practices involve real-time disease prediction, disease risk alerts, and reducing diagnostic errors using several ML algorithms such as SVM, RF, Neural Networks (NN), etc^9,31–34^. Here, our comparative analysis will focus on studies that have used CPD and particularly CBC report features for the screening of hematological disorders.

In a study using CPD^17^, a total of 43 different categories of hematological disorders were identified. RF was observed as the best model with both complete and reduced sets of features, with an overall accuracy of 59% and 57% respectively. The results suggest that a smaller subset of hematological parameters might be sufficient to be exploited as a ‘fingerprint’ of a disease. This study was the first one to demonstrate that successful hematological diagnosis can be made from the results of blood tests alone. Another related study^18^, used blood CPD to predict hematological malignancies. Out of 61 blood parameters, 41 were selected based on point-biserial correlation. The results showed that Artificial Neural Network (ANN) when trained with a selected subset of features, performed the best with 82.8% accuracy and precision, 84.9% recall, and 93.5% AUC. However, the limitations of this study include a lack of external validation and transformation into an end-user tool. A recent study^35^, used selected CBC parameters such as Age, Gender, HB, MCV, MCH, MCHC, and PLT of 346 patients to train different machine learning algorithms for the classification of anemia into its severity levels. The experimental results indicated that the MLP network predominantly gave good recall values across mild and moderate classes which are early and middle stages of the disease. In^36^, Bigorra et al. used leukocyte subpopulation data from the CBC reports of healthy controls, virus-infected patients, and chronic lymphocytic leukemia (CLL) patients for lymphocyte-related diagnosis. The NN model performed the best with 98.7% accuracy in predicting these three categories. Haider et al. in^37^ used CPD from CBC for predictive modeling to differentiate between different types of leukemia. ANN, trained on classical and research CPD from CBC reports, was able to achieve the AUC values of 93.7, 90.5, 80.5, 82.9, 87, and 78.9% for predicting acute myeloid leukemia, chronic myeloid leukemia, acute promyelocytic leukemia, acute lymphoid leukemia, chronic lymphoid leukemia, and other related hematological neoplasms respectively. The findings of this study can be utilized in hematology-oncology department for early leukemia detection, but more clinical and laboratory validation is required for the finalization.

In our study, which has incorporated local CBC reports, the machine learning approach using RF showed significant improvement with an increase in accuracy from 87% with complete set of features to 92% with the selected ‘fingerprint’ of features. A comprehensive feature selection process has been performed, encompassing both statistical and clinical perceptions.

A thorough analysis of the point-biserial correlation coefficients of all predictor features reveals that certain features have weak associations with the target classes. This suggests the presence of a bias in feature selection for assessing target classes during CBC report evaluation. Consequently, the elimination of these weakly associated features has been deemed necessary. Removing features with correlation coefficients below 0.3, representing the most stringent selection criterion in this study, results in a detrimental impact on the diagnostic sensitivity, with a decline from 66% to 51%. However, the selection of the 0.1 threshold, leading to the removal of five features, appears as an optimal choice due to a subtle increase in both accuracy and diagnostic sensitivity compared to the original set of features.

It is worth noting that the evaluation of the suggested features by point-biserial correlation analysis is carried out by three tree-based algorithms i.e., DT, RF, and GBM. These algorithms are then used in the employment of RFE as well, ensuring consistent evaluation criteria for determining feature importance scores. The results of these techniques are integrated with clinically relevant features that are recognized by expert healthcare professionals. This has led to the identification of a more robust and informative set of predictors, surpassing the limitations of relying solely on a single approach. We strongly recommend examining the capacity of ML in the evaluation of various other feature selection techniques.

From our results, it is observed that HB is among the top influential features that are prioritized by all the implemented feature selection techniques. As far as the association of HB with anemia and leukemia is concerned, a lower value of HB is a strong indicator of anemia and should be immediately investigated further. Often anemia can be a result of an existing underlying condition such as leukemia. Clinically, it makes sense that HB achieves the highest importance score in different models. In contrast, the six features that have been removed include demographic features like age and gender, and WBC types like EO, EO%, BASO%, and NEUT% due to their weak association with anemia and leukemia. For example, inclusion of demographic features might lead to a bias and lack of generalizability when applied to populations of different age groups and gender. In consideration of the types of WBCs, basophils and eosinophils mainly take part in allergies, inflammations, and parasitic infections^38^ and therefore, have no involvement in anemia or blood cancers. Neutrophils, on the other hand, are responsible for myeloproliferative disorders (i.e., leukemia), haemorrhages, and myocardial infarctions^39^. However, the absolute count of neutrophils is considered more important for the prediction of leukemia relative to its percentage^10^. Hence, it can be said that removing these features does not lead to exclusion of any important information while training ML models.

The model’s predictions of anemia and leukemia using only the selected ‘fingerprint’ of the CBC report features can be a trustworthy ‘screening’ tool in the clinical workflow. This fingerprint of features was carefully selected using not only statistical approaches but also the practical knowledge of healthcare professionals regarding what in the CBC reports truly influence these conditions. This blend enhances the clinical relevance and interpretability of these features to accurately signal risk and guide further clinical action. The model aims to identify patterns in CBC reports that are highly suggestive of anemia and leukemia. In busy and resource-limited settings, it can be useful to prioritize which CBC reports require immediate expert’s review and attention. This will not only save laboratory resources but also reduce the time from initial blood test to a suspected diagnosis. It is important to note that this model is not designed to provide a definitive diagnosis but to optimize the workflow and ensure that any potentially abnormal CBC results are flagged for the clinician to take the next diagnostic steps. For the model’s translational application, when a CBC is performed, the selected fingerprint of its features can be fed into the ML model, which then flags the report whether it is suspected of anemia, leukemia, or both. For instance, if the patient is suspected of leukemia, an urgent alert will be sent to the workstation of the clinician who ordered the CBC. This way clinicians can prioritize their attention to flagged cases or reports, leading to early follow-up tests and treatment initiation.

Studies mentioned above have encountered the confines of small sample sizes^18,35,36^. The acquisition of labeled and annotated clinical data, due to privacy concerns of patients, is challenging and time-consuming. In this study, we have attempted to surpass this constraint by enriching our original data with synthetic data. The synthetic data has been generated programmatically based on the statistical distributions of the original dataset. Real-world CBC data shows that blood parameters tend to behave distinctively when it comes to anemia, leukemia or the combination of both. While the majority of CBC report features follow the burr distribution, some features exhibit different distributions in different classes. For example, RBC and WBC follow a lognormal distribution in the ‘combination’ class, and HCT follows a gamma distribution in the ‘normal’ and ‘leukemia’ classes. Similarly, PLT and some RBC indices follow a Weibull distribution in certain classes. The burr distribution is a parent distribution with four parameters that can model a range of skewness, making it an appropriate choice for modeling hematological features, particularly when the data displays intricate distributional properties. However, as the features follow different distributions in different target classes, it has been deemed necessary to use a combination of distributions during the generation of random numbers for synthetic data. This has ensured the accurate representation of the underlying patterns and inconsistencies observed in real-world clinical data.

The performance evaluation of the machine learning models trained on hybrid synthetic data for the 14 selected features shows that the RF algorithm achieves exceptional results with 98% accuracy and 97%, 98%, 99%, and 2% macro-averages of precision, recall, specificity, and miss-rate respectively for all four classifications. The ‘anemia’ and ‘combination’ classes have the highest diagnostic sensitivity and lower miss rates while ‘leukemia’ class has the highest miss rate of 3% among all classes. GBM has shown similar results with an accuracy of 97%, followed by DT with 94% accuracy respectively. SVM and MLP (accuracy: 88%) and LR (accuracy: 80%) have performed rather poorly with the highest rates of false negatives. Out of all the classes, ‘leukemia’ class has been frequently observed to be falsely classified as the ‘normal’ class. The unsatisfactory representation of this class is likely due to the inadequate number of instances presented in the original data as the ML models encounter many challenges in unraveling the intrinsic patterns in minority classes often leading to misclassification. During performance evaluation, the overall performance of all models is assessed to determine the top-performing model. However, in a clinical setting, the most important metric would be the recall or true positive rate of the classification model, as the misclassification of diseased individuals as normal would have serious downstream repercussions. Therefore, during external validation, only recall of the model is evaluated.

External validation of the model on a secondary dataset of 270 patients from Iraq resulted in an overall accuracy of 74%, with poor recall for the Normal and Anemia classes, where many instances are misclassified as the Leukemia and/or Combination class. Recall for the Leukemia and Combination class are quite high, which shows that the model is over-fit for these 2 classes. This may have occurred due to feature correlation bias in the training data, which may have strongly correlated features for the leukemic and combination class. Such strong correlation patterns may not be present in the external validation dataset. This indicates that the final RF model is not generalizable on external data collected from different demographic or ethnic backgrounds. Poor performance during external validation may also be attributed to the use of synthetic data that was generated using a small-sized and class-imbalanced dataset. For the efficient learning of underlying patterns and existing biases to generate better quality synthetic data, an adequate size of original data is required. This study proposes and tests a methodology that utilizes synthetic data generation to address the limitation of scarce labelled clinical data. While the training results were promising, the lack of generalizability of the final model during external validation evidences the requirement of extensive and real clinical data for the development of an efficient ML-based decision support system for the screening of anemia and leukemia. Despite the modest results, the incorporation of external validation in our study is a valuable contribution, as this step is seldom reported in the existing literature on predicting blood disorders.

Further limitations and future recommendations for the study are also discussed. Our study mainly focuses on predictive modeling using local CBC report features and does not consider additional blood parameters. Investigation of features such as patient symptoms along with blood parameters should be incorporated in future studies. This research can also be extended further to predict the subtypes and causes of anemia and leukemia or include other blood disorders such as blood infections, thalassemia, lymphoma, etc. Additionally, game theoretic approaches like SHAP can be integrated in the final model to provide explainable outputs of individual cases. This will provide clinicians insights on the most significant CBC report features for each patient, resulting in more personalized healthcare. We also strongly suggest exploring other methods for generating synthetic data. For this purpose, deep learning approaches i.e., Generative Adversarial Networks (GANs)^40^ and Variational Autoencoders (VAEs)^41^ can also be employed. Particularly, techniques like Copula models that account for inter-variable dependencies during data augmentation should be experimented upon^42^, as the current study used each feature independently to model new instances.

## Conclusion

This research presents a novel approach of using a ‘fingerprint’ of features of local CBC reports and hybrid synthetic data to train ML models for the screening of two common blood disorders – anemia and leukemia. Hybrid synthetic data addresses the issue of the small sample size and appears to be a promising alternative for real-world data. Exceptional performance has been observed by the RF algorithm with the highest accuracy, precision, recall, specificity, and lowest miss-rate relative to other ML algorithms. However, external validation of the model on a dataset of patients from Iraq achieved an accuracy of 74%, indicating that the model is not generalizable on a different demographic cohort. The study highlights the crucial need of an extensive, diverse, real dataset for the development of generalizable and efficient ML-based screening system. In the future, we are planning to validate our results with external data and transform the suggested process into a systematic and user-friendly smart tool for the end-users. However, further research and validation are necessary for this tool to be used in clinical practices.

## Supplementary Information

Below is the link to the electronic supplementary material.

Supplementary Material 1

Supplementary Material 2

## Data Availability

Data and code used to train the models may be made available upon request from the corresponding author (mahmood.ul.hassan@ki.se) after acceptance.
