# Supplementary material for: Machine learning-based models for screening of anemia and leukemia using features of complete blood count reports
Source: Sci Rep. 2025 Sep 29;15:33333. doi: 10.1038/s41598-025-21279-w (PMC12480587; doi:10.1038/s41598-025-21279-w)
Supplement: Supplementary file 1 — Supplementary Material 1 [file 41598_2025_21279_MOESM1_ESM.pdf]

Table S1 Performance metrics of the six ML models trained with a random seed value of 0.

Model	Accuracy	Classes	Precision	Recall	Specificity
**DT**	0.948 ± 0.010	Normal	0.926 ± 0.029	0.955 ± 0.015	0.975 ± 0.011
		Anemia	0.966 ± 0.012	0.965 ± 0.018	0.980 ± 0.004
		Leukemia	0.934 ± 0.020	0.913 ± 0.030	0.981 ± 0.006
		Combination	0.974 ± 0.010	0.965 ± 0.015	0.989 ± 0.004
**RF**	0.975 ± 0.006	Normal	0.964 ± 0.017	0.965 ± 0.007	0.988 ± 0.006
		Anemia	0.990 ± 0.006	0.988 ± 0.011	0.997 ± 0.002
		Leukemia	0.953 ± 0.014	0.958 ± 0.019	0.986 ± 0.005
		Combination	0.990 ± 0.009	0.986 ± 0.013	0.996 ± 0.004
**GBM**	0.971 ± 0.010	Normal	0.949 ± 0.032	0.985 ± 0.007	0.983 ± 0.012
		Anemia	0.979 ± 0.007	0.986 ± 0.013	0.984 ± 0.002
		Leukemia	0.969 ± 0.010	0.947 ± 0.036	0.991 ± 0.003
		Combination	0.991 ± 0.007	0.971 ± 0.015	0.996 ± 0.003
**SVM**	0.876 ± 0.019	Normal	0.825 ± 0.034	0.887 ± 0.040	0.940 ± 0.016
		Anemia	0.803 ± 0.033	0.977 ± 0.013	0.929 ± 0.015
		Leukemia	0.906 ± 0.026	0.746 ± 0.076	0.976 ± 0.009
		Combination	0.981 ± 0.009	0.890 ± 0.043	0.992 ± 0.004
**LR**	0.804 ± 0.021	Normal	0.751 ± 0.042	0.804 ± 0.034	0.914 ± 0.020
		Anemia	0.719 ± 0.023	0.824 ± 0.041	0.906 ± 0.007
		Leukemia	0.789 ± 0.030	0.678 ± 0.051	0.945 ± 0.012
		Combination	0.943 ± 0.009	0.885 ± 0.044	0.977 ± 0.004
**MLP**	0.880 ± 0.028	Normal	0.866 ± 0.058	0.835 ± 0.081	0.957 ± 0.025
		Anemia	0.820 ± 0.054	0.894 ± 0.064	0.941 ± 0.023
		Leukemia	0.906 ± 0.048	0.868 ± 0.098	0.971 ± 0.017
		Combination	0.964 ± 0.015	0.942 ± 0.022	0.979 ± 0.007

Table S2 Performance metrics of the six ML models trained with a random seed value of 1.

Model	Accuracy	Classes	Precision	Recall	Specificity
**DT**	0.943 ± 0.009	Normal	0.921 ± 0.020	0.953 ± 0.020	0.974 ± 0.008
		Anemia	0.961 ± 0.023	0.954 ± 0.023	0.983 ± 0.007
		Leukemia	0.923 ± 0.018	0.905 ± 0.025	0.977 ± 0.006
		Combination	0.966 ± 0.020	0.955 ± 0.017	0.985 ± 0.009
**RF**	0.976 ± 0.008	Normal	0.973 ± 0.013	0.960 ± 0.018	0.991 ± 0.004
		Anemia	0.994 ± 0.007	0.990 ± 0.008	0.998 ± 0.002
		Leukemia	0.945 ± 0.019	0.962 ± 0.026	0.983 ± 0.006
		Combination	0.988 ± 0.008	0.987 ± 0.010	0.995 ± 0.004
**GBM**	0.972 ± 0.016	Normal	0.946 ± 0.017	0.987 ± 0.013	0.982 ± 0.006
		Anemia	0.979 ± 0.007	0.992 ± 0.007	0.994 ± 0.002
		Leukemia	0.977 ± 0.013	0.949 ± 0.025	0.993 ± 0.004
		Combination	0.989 ± 0.003	0.975 ± 0.006	0.984 ± 0.001
**SVM**	0.875 ± 0.011	Normal	0.827 ± 0.018	0.887 ± 0.022	0.941 ± 0.007
		Anemia	0.802 ± 0.028	0.985 ± 0.014	0.928 ± 0.013
		Leukemia	0.899 ± 0.019	0.744 ± 0.037	0.975 ± 0.005
		Combination	0.981 ± 0.006	0.884 ± 0.022	0.992 ± 0.002
**LR**	0.804 ± 0.007	Normal	0.756 ± 0.018	0.802 ± 0.043	0.918 ± 0.007
		Anemia	0.713 ± 0.022	0.832 ± 0.029	0.902 ± 0.012
		Leukemia	0.804 ± 0.048	0.671 ± 0.035	0.949 ± 0.017
		Combination	0.935 ± 0.017	0.887 ± 0.029	0.973 ± 0.008
**MLP**	0.867 ± 0.010	Normal	0.832 ± 0.040	0.860 ± 0.040	0.944 ± 0.018
		Anemia	0.795 ± 0.031	0.903 ± 0.018	0.931 ± 0.014
		Leukemia	0.892 ± 0.052	0.750 ± 0.053	0.971 ± 0.018
		Combination	0.951 ± 0.018	0.934 ± 0.024	0.979 ± 0.008

Table S3 Performance metrics of the six ML models trained with a random seed value of 42.

Model	Accuracy	Classes	Precision	Recall	Specificity
**DT**	0.947 ± 0.008	Normal	0.913 ± 0.021	0.967 ± 0.018	0.971 ± 0.008
		Anemia	0.957 ± 0.025	0.959 ± 0.022	0.987 ± 0.008
		Leukemia	0.945 ± 0.014	0.899 ± 0.040	0.984 ± 0.005
		Combination	0.971 ± 0.011	0.957 ± 0.016	0.987 ± 0.005
**RF**	0.980 ± 0.007	Normal	0.975 ± 0.015	0.978 ± 0.014	0.992 ± 0.005
		Anemia	0.990 ± 0.008	0.986 ± 0.010	0.997 ± 0.002
		Leukemia	0.964 ± 0.019	0.968 ± 0.014	0.989 ± 0.006
		Combination	0.988 ± 0.006	0.986 ± 0.004	0.995 ± 0.002
**GBM**	0.971 ± 0.008	Normal	0.938 ± 0.020	0.984 ± 0.007	0.979 ± 0.007
		Anemia	0.975 ± 0.012	0.988 ± 0.007	0.993 ± 0.004
		Leukemia	0.972 ± 0.010	0.937 ± 0.023	0.992 ± 0.003
		Combination	0.994 ± 0.003	0.973 ± 0.008	0.997 ± 0.001
**SVM**	0.879 ± 0.021	Normal	0.830 ± 0.026	0.891 ± 0.025	0.942 ± 0.010
		Anemia	0.808 ± 0.047	0.983 ± 0.013	0.931 ± 0.020
		Leukemia	0.904 ± 0.031	0.746 ± 0.036	0.976 ± 0.008
		Combination	0.983 ± 0.009	0.893 ± 0.036	0.993 ± 0.004
**LR**	0.816 ± 0.024	Normal	0.759 ± 0.043	0.827 ± 0.031	0.915 ± 0.022
		Anemia	0.727 ± 0.040	0.840 ± 0.037	0.907 ± 0.016
		Leukemia	0.836 ± 0.044	0.686 ± 0.012	0.959 ± 0.013
		Combination	0.942 ± 0.015	0.889 ± 0.045	0.976 ± 0.006
**MLP**	0.879 ± 0.018	Normal	0.854 ± 0.051	0.844 ± 0.086	0.952 ± 0.023
		Anemia	0.801 ± 0.069	0.897 ± 0.069	0.931 ± 0.035
		Leukemia	0.898 ± 0.030	0.816 ± 0.050	0.972 ± 0.010
		Combination	0.970 ± 0.010	0.941 ± 0.021	0.987 ± 0.004
