# Supplementary material for: Machine learning-based models for screening of anemia and leukemia using features of complete blood count reports
Source: Sci Rep. 2025 Sep 29;15:33333. doi: 10.1038/s41598-025-21279-w (PMC12480587; doi:10.1038/s41598-025-21279-w)
Supplement: Supplementary file 2 — Supplementary Material 2 [file 41598_2025_21279_MOESM2_ESM.pdf]

Synthetic Data Generation

The dataset used in this study is small in size with only 287 instances. It is difficult to obtain labeled and annotated medical data due to privacy and ethical concerns. Therefore, synthetic data are generated in this study to improve the resilience and flexibility of the models [1], [2]. Synthetic data are generated based on the statistical distributions followed by the selected CBC features for each target class using EasyFit 5.6 Professional [3]. Lognormal, gamma, Weibull, and burr distributions are selected to model the continuous and non-negative blood parameters based on literature support [4], [5], [6]. The details of these probability distributions are given in the Appendix. The validation of the goodness-of-fit of these distributions is achieved by Kolmogorov-Smirnov and Anderson Darling tests at an alpha level of 0.05. If the calculated statistical quantity of each of these tests is smaller than the critical value for that test, it indicates that the applied distribution matches the sample data for that particular CBC report feature. Using the best-fitted distributions, new data points are then generated using a random number generator algorithm in EasyFit software. These new synthetic data points retain the distributional properties of the original data. The synthetic data for each target class are then combined with the original data points to generate a 'hybrid' synthetic dataset consisting of 2287 instances.

Theoretical Probability Distributions

Several investigations have explored the appropriate probability distributions for modeling blood parameters. Studies have highlighted the suitability of lognormal, gamma, and Weibull distributions for various applications. For instance, analyzing blood cell counts and percentages frequently utilizes gamma, Weibull, and lognormal distributions. In 2016, Shrestha et al. employed a modeling technique to analyze the residual survival data of the biotin-labeled RBCs, incorporating models based on these three distributions [4]. Another study investigating the interaction between HIV-1 and WBCs, utilized a gamma distribution to represent individual cellular variation in delay times between the initial infection and infected cell creation [7]. Furthermore, literature reports research on the modified Weibull distribution of relaxation time for human blood, analyzed using statistical methods to study the dielectric characteristics of blood cells, highlighting the potential of dielectric spectroscopy as a non-invasive tool for

leukemia diagnosis [5]. The details of these probability distributions are explained in the section below.

Lognormal Distribution

Lognormal distribution is frequently used in biological and financial areas of research to model the right-skewed data. It is a two or three parameter distribution with μ, σ, and γ as the shape, scale, and location parameters respectively. γ=0 yields the two-parameter lognormal distribution [8]. Equation (S1) gives the probability density function of lognormal distribution.

$$f(x) = \frac{exp\left[-\frac{1}{2}\left(\frac{ln(x-\gamma)-\mu}{\sigma}\right)^2\right]}{(x-\gamma)\,\sigma\,\sqrt{2\pi}} \tag{S1}$$

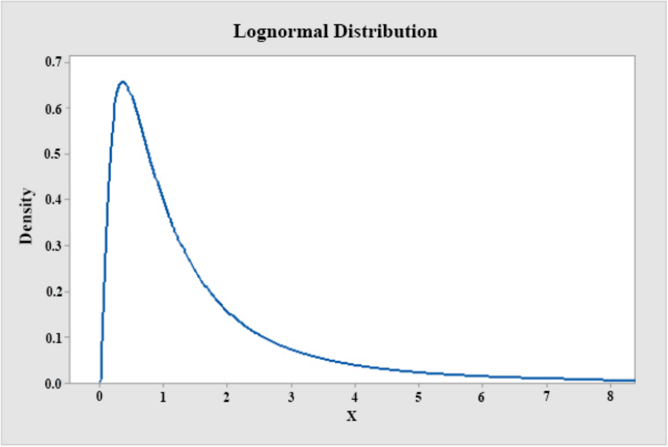

Figure S1: Lognormal Distribution Plot

Gamma Distribution

Gamma distribution also models right-skewed data, particularly in the fields of science, business, and engineering [9]. This distribution has three parameters: shape (α), scale (β), and location (γ). γ=0 yields the two-parameter gamma distribution. The symbol 'Γ' in the probability density function of gamma distribution given in (S2) represents gamma function.

$$f(x) = \frac{(x-\gamma)^{\alpha-1}}{\beta^{\alpha}\Gamma(\alpha)}\,exp\left(-\frac{x-\gamma}{\beta}\right) \tag{S2}$$

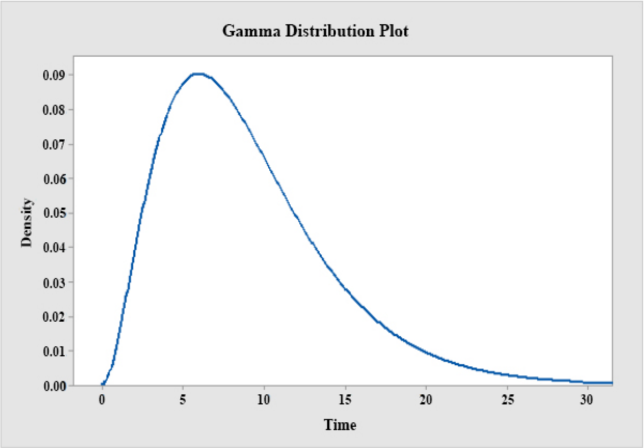

Figure S2: Gamma Distribution Plot

<u>Weibull Distribution</u>

Weibull distribution is adaptable to varying conditions and models both right and left-skewed data [10]. This distribution describes the probability distribution of non-negative and continuous data. Weibull distribution function is quite versatile and flexible due to which it fits a variety of shapes. It is mainly utilized in medical studies, quality control, reliability analysis, etc. It has two variations with two and three parameters of shape (α), scale (β), and threshold (γ). γ=0 yields the two-parameter Weibull distribution (S3).

$$f(x) = \frac{\alpha}{\beta}\left(\frac{x-\gamma}{\beta}\right)^{\alpha-1} exp\left(-\left(\frac{x-\gamma}{\beta}\right)^{\alpha}\right) \quad \text{(S3)}$$

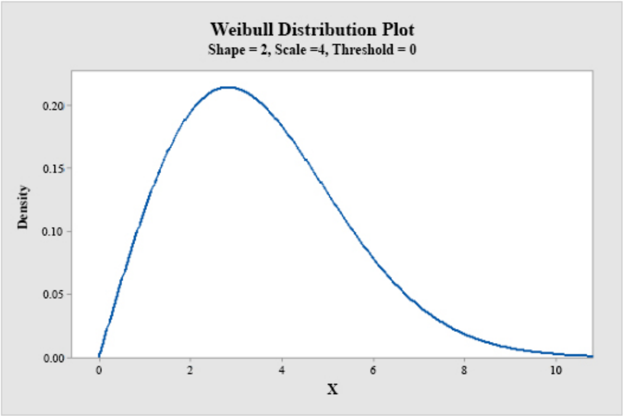

Figure S3: Weibull Distribution Plot

Burr Distribution

Burr distribution models a broad set of skewness and kurtosis. It is the parent distribution of many other distributions such as Weibull, exponential, logistic, etc. It has three or four parameters. k and α are the shape parameters while β and γ are the scale and location parameters respectively [11]. γ=0 yields the three-parameter burr distribution. The probability density function of burr distribution is given in (S4).

$$f(x) = \frac{\alpha k \left(\frac{x-\gamma}{\beta}\right)^{\alpha-1}}{\beta\left(1+\left(\frac{x-\gamma}{\beta}\right)^{\alpha}\right)^{k+1}} \tag{S4}$$

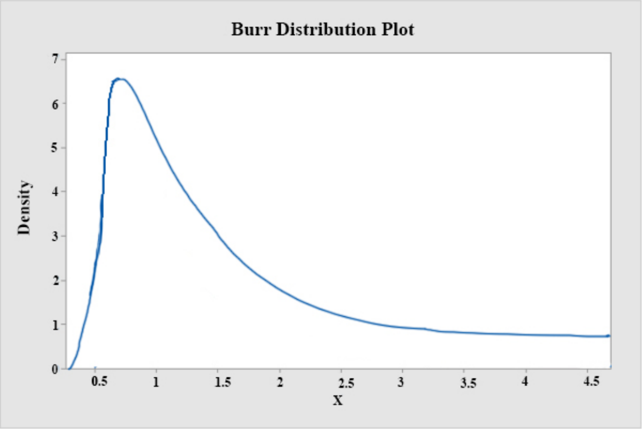

Figure S4: Burr Distribution Plot

P-P Plots

A Probability-Probability (P-P) plot is a graphical statistical tool that compares the empirical distribution of the given data to that of a theoretical probability distribution [12]. Alignment of the data points with the diagonal line on the p-p plot indicates a goodness-of-fit of the data with that theoretical probability distribution. While a deviation from the diagonal line refers to a deviation of the empirical data distribution from the theoretical distribution [13]. Interpretation of the p-p plots for the above-mentioned distributions has led to the selection of the best fitted theoretical probability distribution for each of the selected CBC report features.

Validation of the Goodness-of-fit

After the selection of the best-fitted distributions, validation has been done by analyzing Kolmogorov-Smirnov (KS) and Anderson-Darling tests at an alpha level of 0.05. Both these tests are used to test the goodness-of-fit of the theoretical distributions that have been selected. KS test gives more weight to the center of the distribution whereas the Anderson-Darling test takes into account the tails of the distribution. Both of these tests evaluate the following null (H_{0}) and alternate hypothesis (H_A) respectively:

H_0 = The given CBC parameter follows the selected probability distribution

H_A = The given CBC parameter does not follow the selected probability distribution

The best fitted distributions are validated if the null hypothesis is not rejected at an alpha level of 0.05.

Random Number Generation

Random numbers have been generated for each of the CBC features for the four target classes. This has been done using the random number generator algorithm of the EasyFit software, keeping in mind the parameters of the best-fitted probability distributions followed by the CBC features. The number of random numbers to be generated has been set to 500 for each class.

Results of Synthetic Data Generation

To generate synthetic data, the dataset has been split on the basis of the four target classes – normal, anemia, leukemia, and combination. After fitting the selected theoretical probability distributions, the best-fitted distributions have been selected on evaluating the P-P plots, KS, and Anderson Darling tests. For all the target classes, the stated null hypothesis (The given CBC parameter follows the selected probability distribution) has been accepted for the following probability distribution parameters given in Tables S1 to S4. The P-P plots and probability distribution parameters for the best-fitted distribution for each of the target class are given below.

P-P Plots and Distribution Parameters

P-P Plots and Distribution parameters for "Normal" class are illustrated in Figure S5 and Table S1 below.

(A)
HB | Burr (4P)

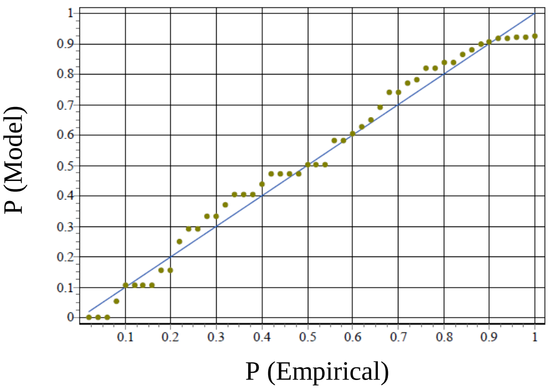

(B)
RBC | Burr (4P)

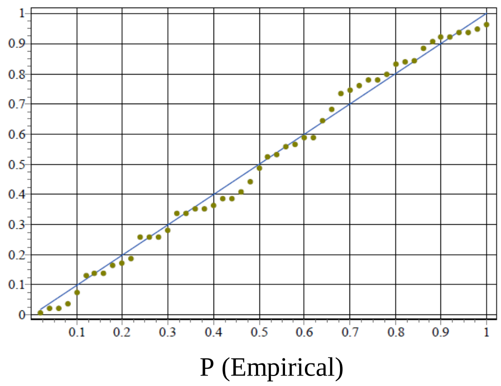

(C)
MONO | Burr (4P)

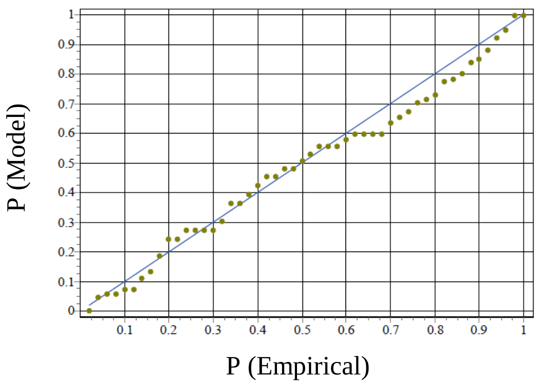

(D)
LYM% | Burr (4P)

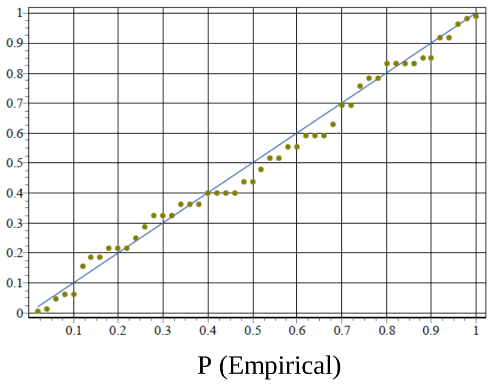

(E)
WBC | Burr (3P)

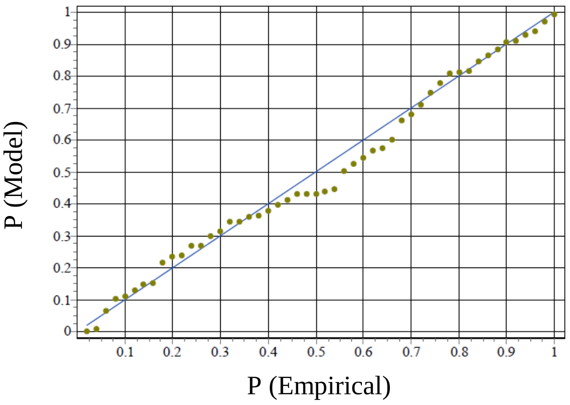

(F)
MCH | Burr (3P)

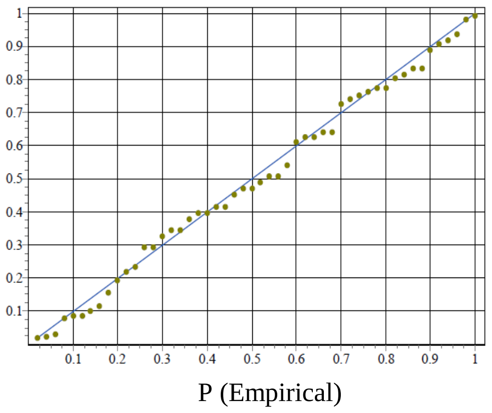

(G)
MCHC | Burr (3P)

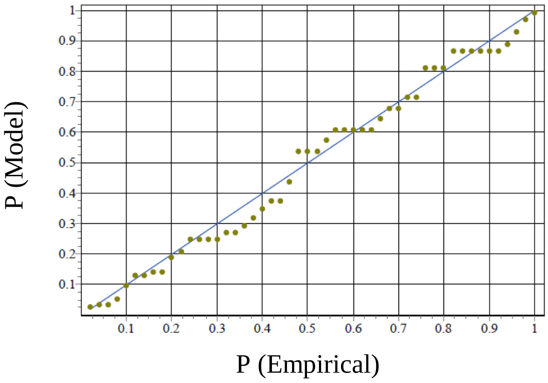

(H)
NEUT | Burr (3P)

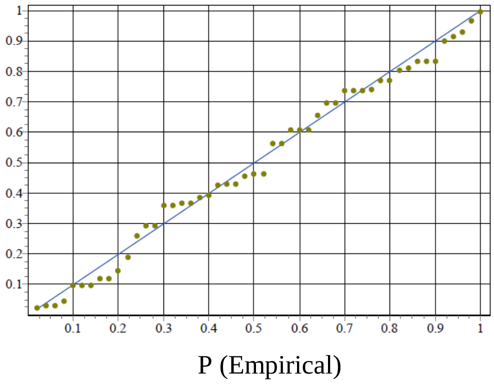

(I)
LYM | Burr (3P)

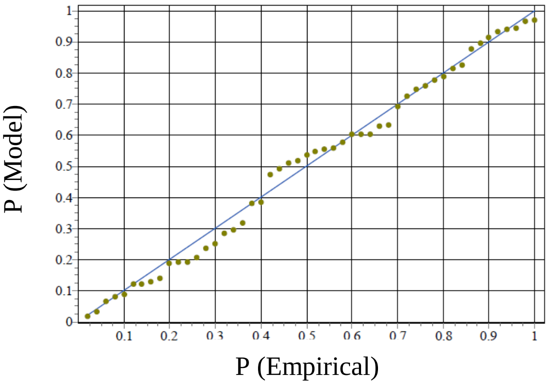

(J)
BASO | Burr (3P)

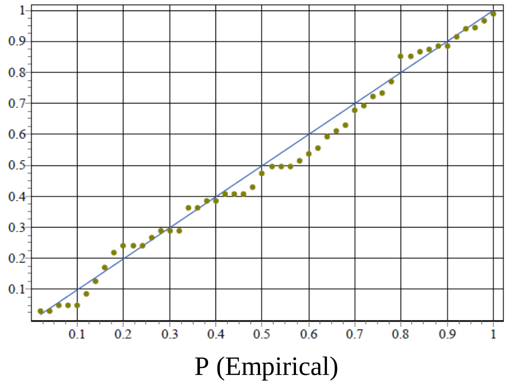

(K)
MONO% | Burr (3P)

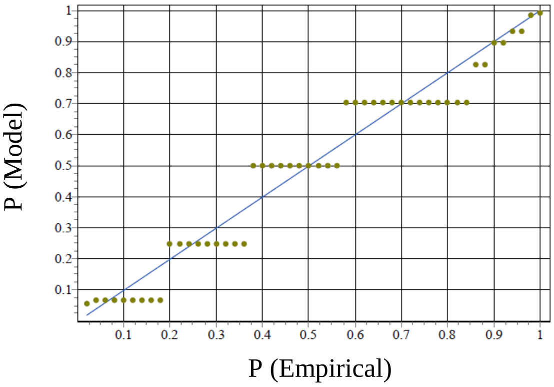

(L)
MCV | Weibull (2P)

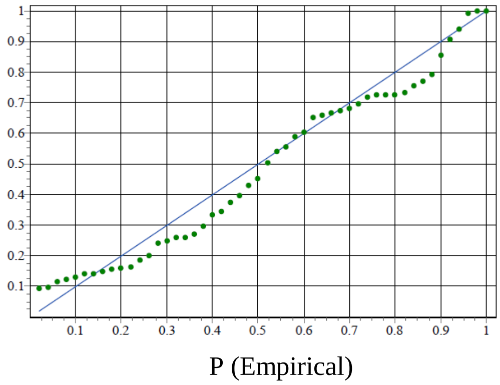

(M)
PLT | Weibull (2P)

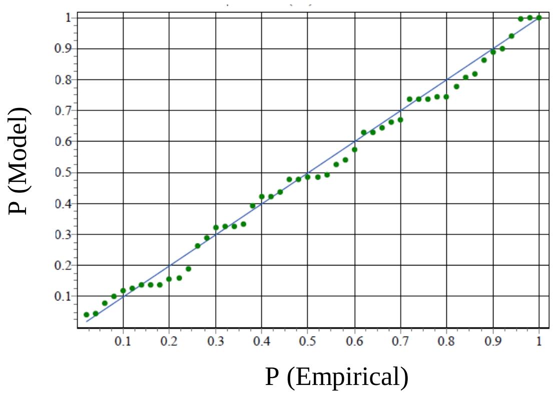

(N)
HCT | Gamma (2P)

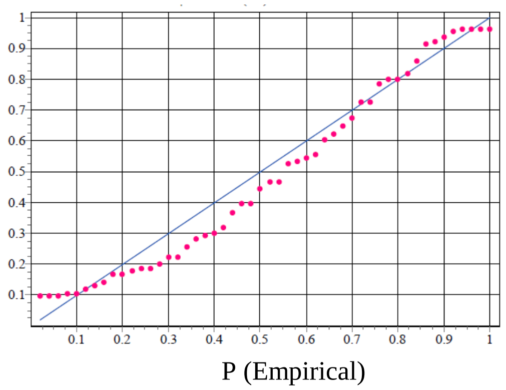

Figure S5: P-P plots for best-fitted distributions of features for 'normal' class

Table S1: Parameters of probability distributions followed by CBC features for 'normal' class

Features	Distribution	Parameters
HB	Burr (4P)	**k** = 4.6237×10^7| α = 1.0208 | β = 5.5417×10^7 | γ = 11.998
RBC		**k** = 1198.4 | α = 1.6547 | β = 69.747 | γ = 3.8068
MONO		**k** = 0.4841 | α = 4.9427 | β = 0.2796 | γ = 0.0243
LYM%		**k** = 23.682 | α = 2.5039 | β = 90.309 | γ = 7.3918
WBC	Burr (3P)	**k** = 0.5778 | α = 6.6872 | β = 6.827
MCH		**k** = 0.7667 | α = 23.080 | β = 28.682
MCHC		**k** = 49.794 | α = 33.543 | β = 38.488
NEUT		**k** = 1.7615 | α = 7.8354 | β = 5.0186
LYM		**k** = 0.5389 | α = 6.9134 | β = 1.8617
BASO		**k** = 16.708 | α = 1.4874 | β = 2.0277
MONO%		**k** = 0.7698 | α = 5.3333 | β = 4.6542
MCV	Weibull (2P)	α = 19.010 | β = 89.148
PLT		α = 5.8389 | β = 268.21
HCT	Gamma (2P)	α = 103.63 | β = 0.3925

P-P Plots and Distribution parameters for "anemia" class are illustrated and mentioned in Figure S6 and Table S2 respectively.

(A)
HCT | Burr (4P)

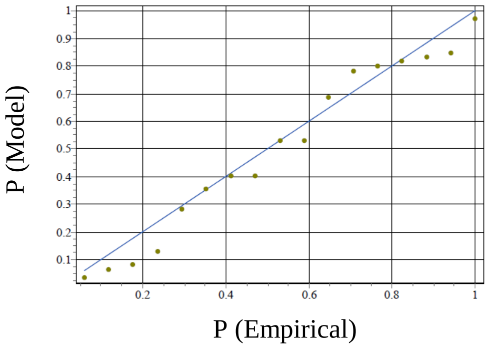

(B)
MCHC | Burr (4P)

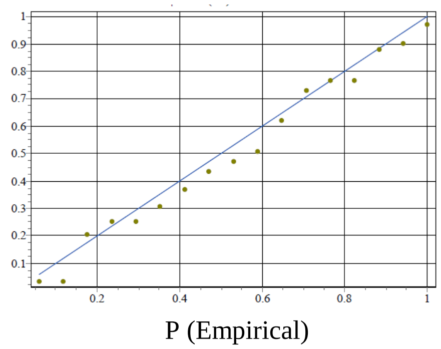

(C)
WBC | Burr (3P)

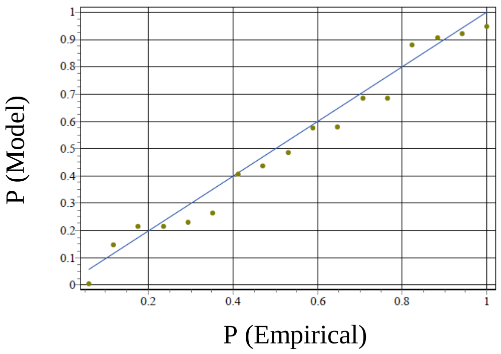

(D)
RBC | Burr (3P)

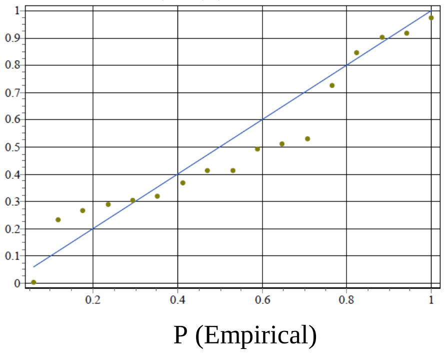

(E)
HB | Burr (3P)

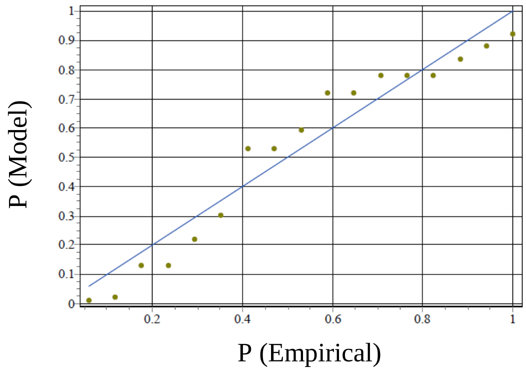

(F)
MCH | Burr (3P)

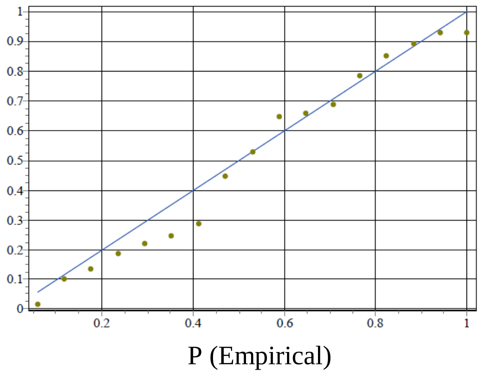

(G)
NEUT | Burr (3P)

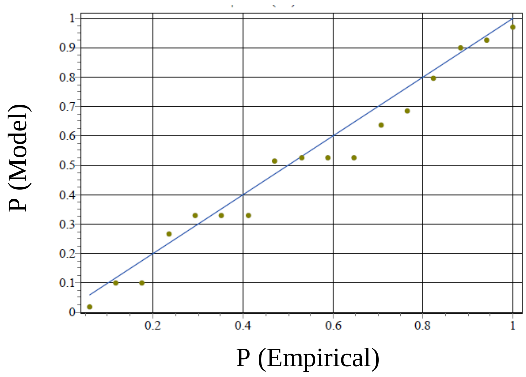

(H)
LYM | Burr (3P)

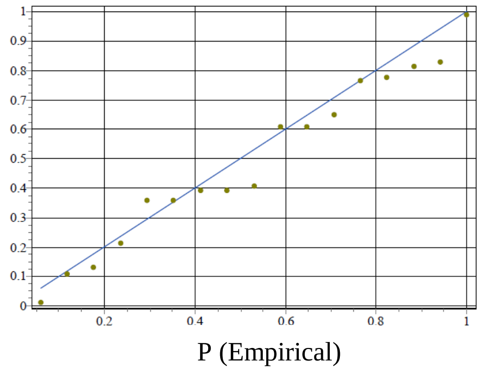

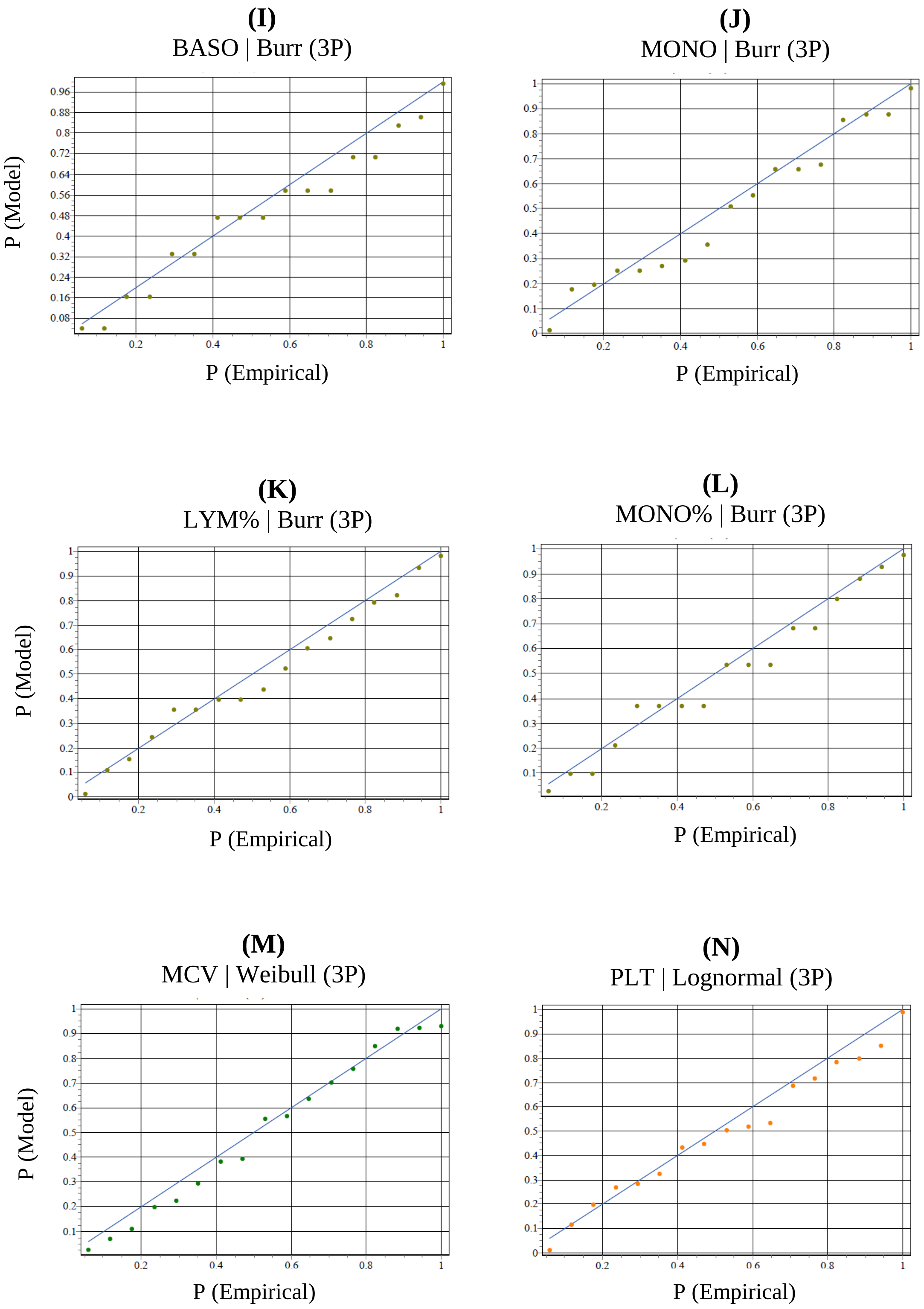

Figure S6: P-P plots for best-fitted distributions of features for 'anemia' class

Table S2: Parameters of probability distributions followed by CBC features for 'anemia' class

Features	Distribution	Parameters
HCT	Burr (4P)	**k** = 2816.7 | α = 880.03 | β = 1636.4 | γ = -1587.2
MCHC		**k** = 22.094 | α = 9.8540 | β = 12.827 | γ = 23.335
WBC	Burr (3P)	**k** = 557.09 | α = 6.4297 | β = 21.162
RBC		**k** = 1.5991 | α = 12.885 | β = 4.1379
HB		**k** = 1227.7 | α = 19.953 | β = 16.082
MCH		**k** = 322.75 | α = 11.329 | β = 47.619
NEUT		**k** = 0.5548 | α = 14.259 | β = 4.1828
LYM		**k** = 0.5586 | α = 7.0591 | β = 1.9741
BASO		**k** = 0.3254 | α = 4.1503 | β = 0.0322
MONO		**k** = 8.9615 | α = 2.7552 | β = 1.0376
LYM%		**k** = 44.504 | α = 3.6568 | β = 91.838
MONO%		**k** = 3.0978 | α = 3.0720 | β = 9.0812
MCV	Weibull (3P)	α = 3.3976 | β = 25.583 | γ = 60.264
PLT	Lognormal (3P)	σ = 0.4239 | μ = 4.8216 | γ = 116.49

P-P Plots and Distribution parameters for "leukemia" class are illustrated and mentioned in Figure S7 and Table S3 respectively.

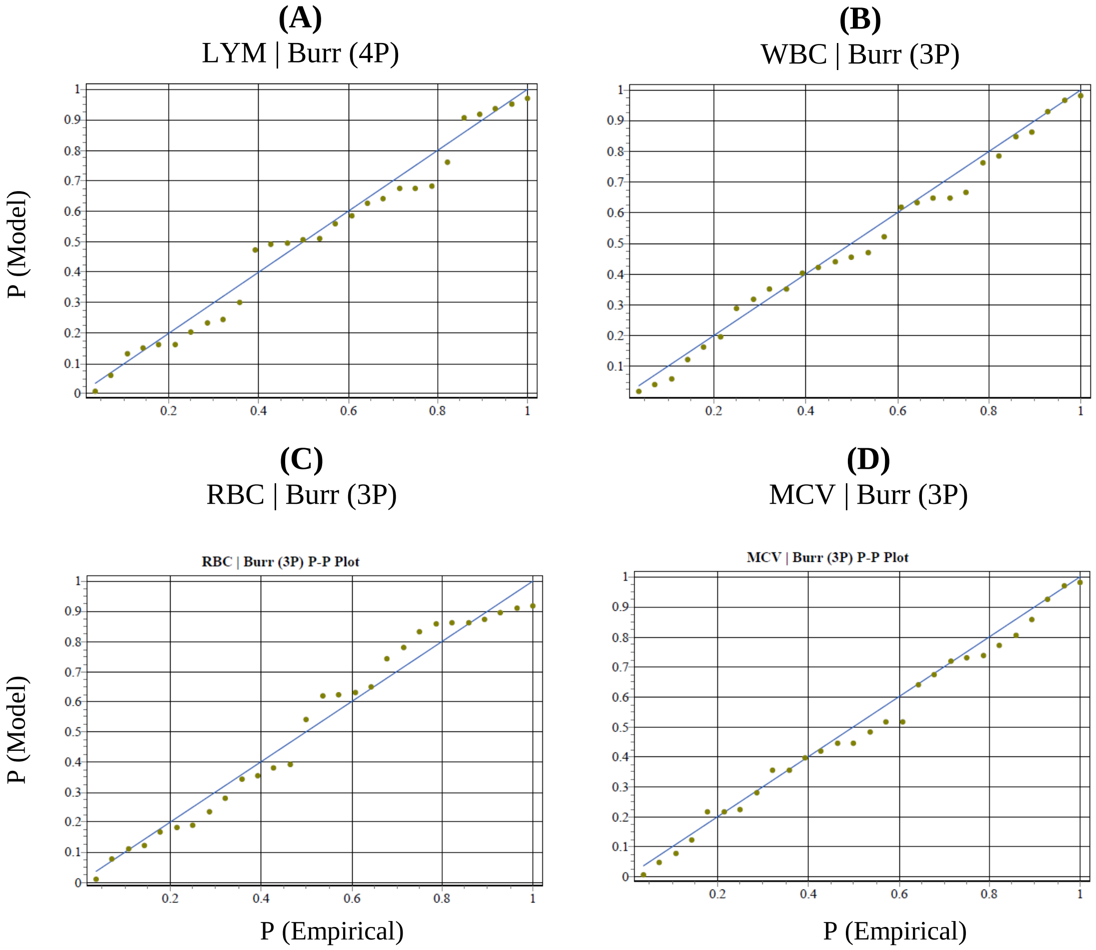

(E)
PLT | Burr (3P)

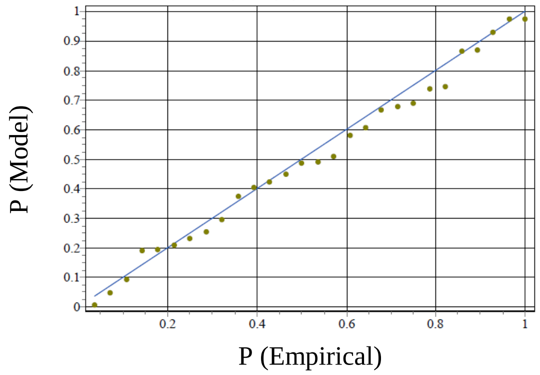

(F)
BASO | Burr (3P)

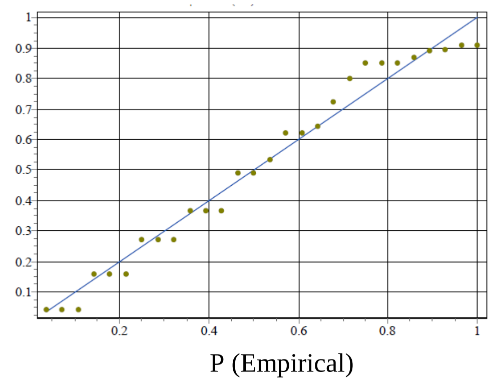

(G)
MONO | Burr (3P)

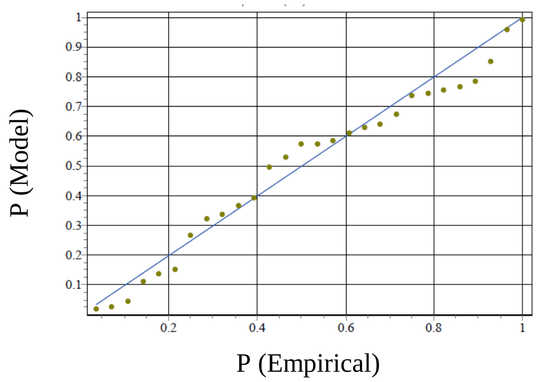

(H)
LYM% | Burr (3P)

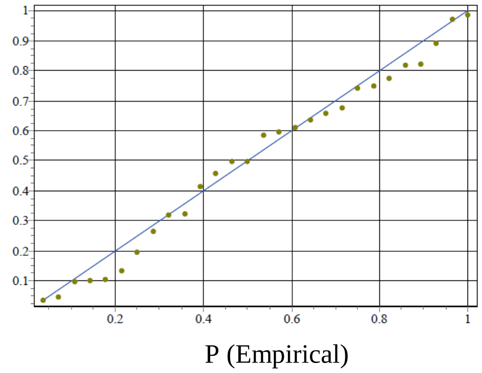

(I)
MONO% | Burr (3P)

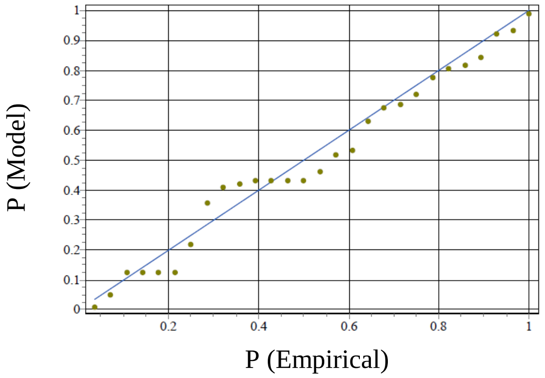

(J)
MCH | Burr (3P)

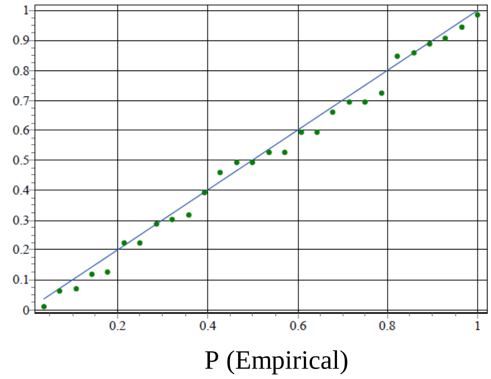

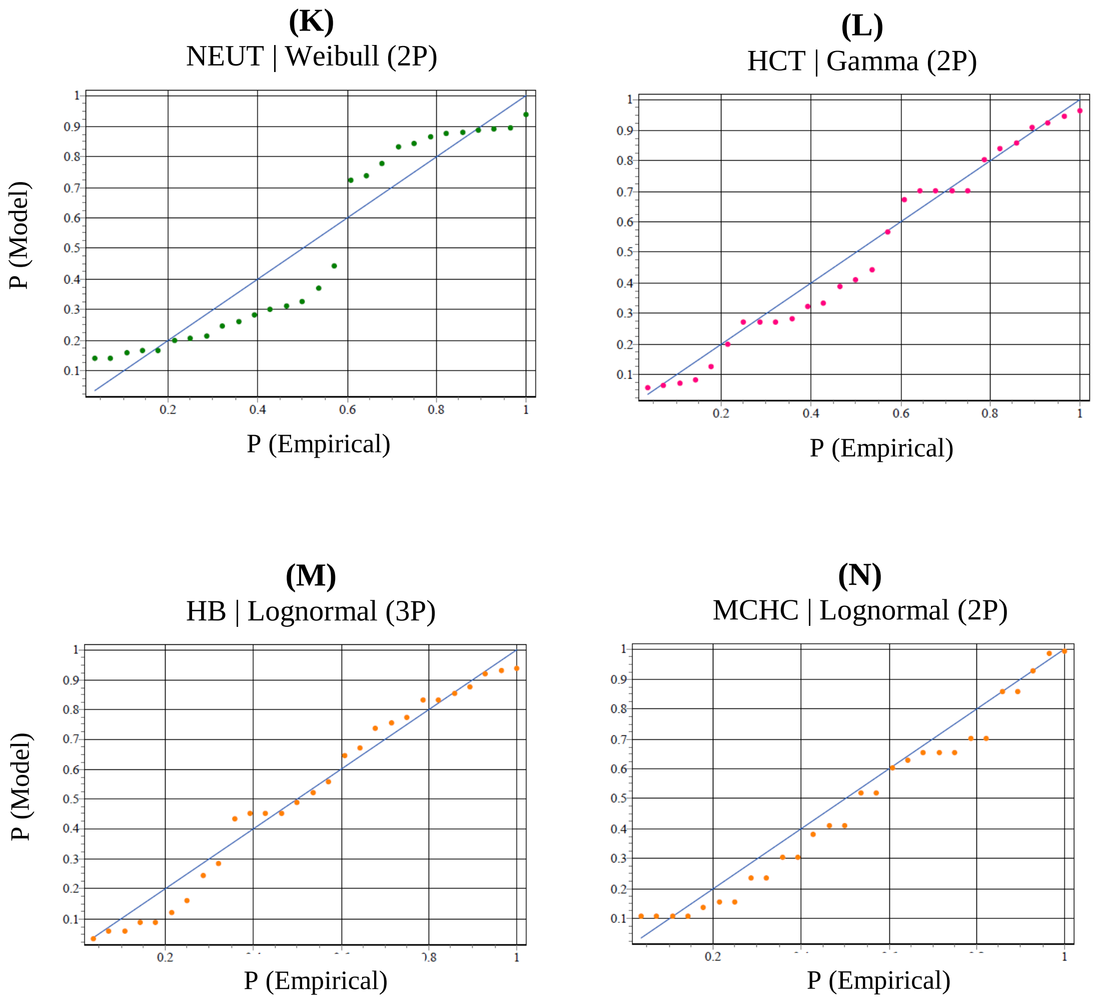

Figure S7: P-P plots for best-fitted distributions of features for ‘leukemia’ class

Table S3: Parameters of probability distributions followed by CBC features for ‘leukemia’ class

Features	Distribution	Parameters
LYM	Burr (4P)	**k** = 0.5203 | α = 3.4683 | β = 1.2958 | γ = 0.31137
WBC	Burr (3P)	**k** = 2.5247 | α = 3.4273 | β = 8.6232
RBC		**k** = 0.2818 | α = 27.650 | β = 4.0336
MCV		**k** = 0.6093 | α = 32.974 | β = 83.857
PLT		**k** = 3.7782 | α = 2.4797 | β = 392.65
BASO		**k** = 0.2606 | α = 2.3505 | β = 0.0207
MONO		**k** = 1.9240 | α = 1.7306 | β = 0.6861
LYM%		**k** = 2.3330 | α = 4.0126 | β = 39.213
MONO%		**k** = 1.1602 | α = 2.3740 | β = 7.3107
MCH	Weibull (3P)	α = 4.9769 | β = 10.910 | γ = 19.717
NEUT	Weibull (2P)	α = 0.8436 | β = 17.449
HCT	Gamma (2P)	α = 124.04 | β = 0.3160
HB	Lognormal (3P)	σ = 0.6071 | μ = 0.6075 | γ = 11.296
MCHC	Lognormal (2P)	σ = 0.0417 | μ = 3.5388

The P-P Plots and Distribution parameters for “combination” class are illustrated and mentioned in Figure S8 and Table S4 respectively.

(A)
HB | Burr (4P)

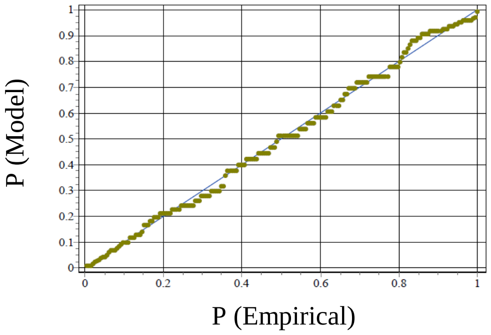

(B)
NEUT | Burr (3P)

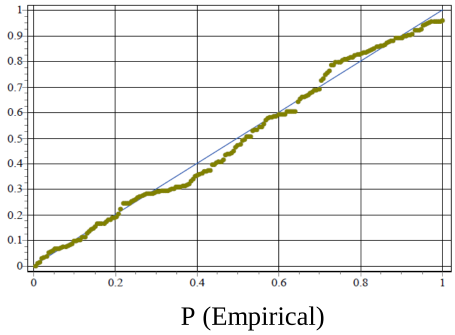

(C)
MCV | Burr (3P)

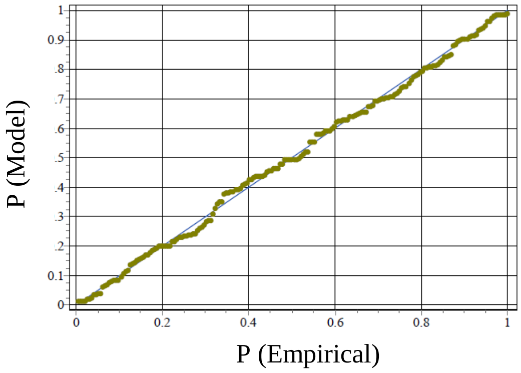

(D)
MCH | Burr (3P)

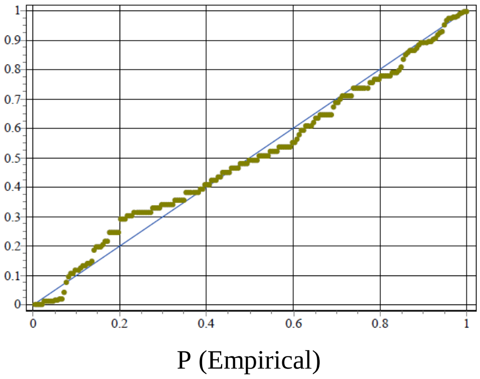

(E)
MCHC | Burr (3P)

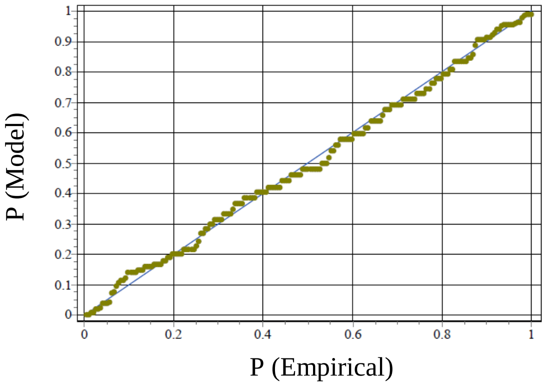

(F)
BASO | Burr (3P)

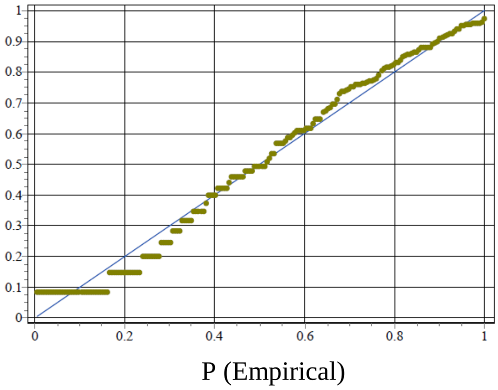

(G)

HCT | Weibull (2P)

P (Model)

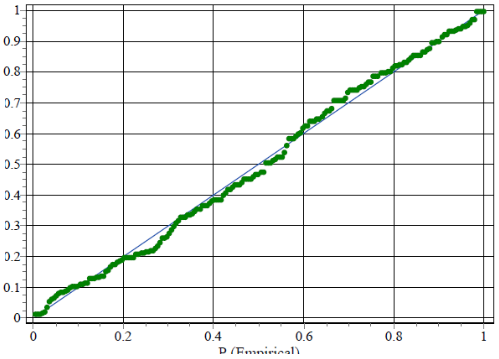

P (Empirical)

(H)

PLT | Weibull (2P)

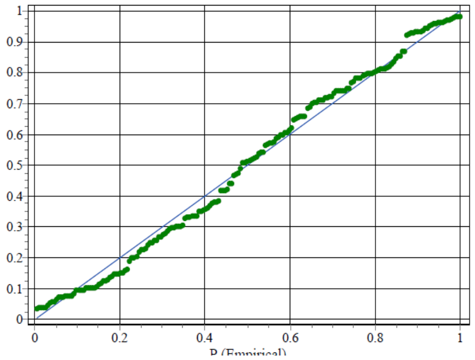

P (Empirical)

(I)

LYM% | Weibull (2P)

P (Model)

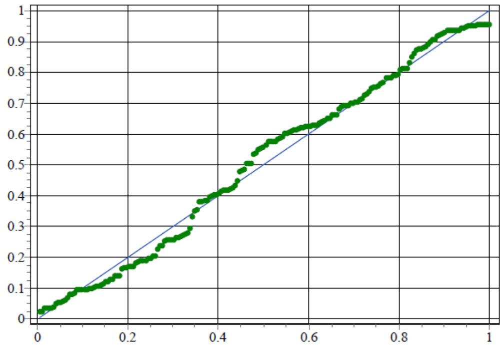

P (Empirical)

(J)

MONO% | Gamma (3P)

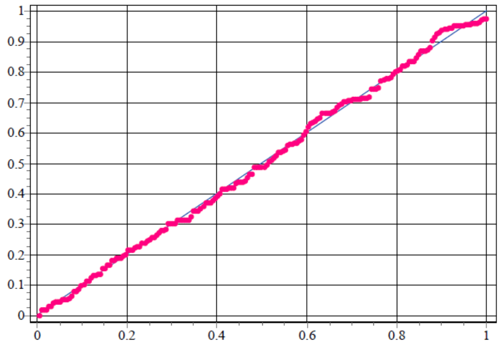

P (Empirical)

(K)

WBC | Lognormal (3P)

P (Model)

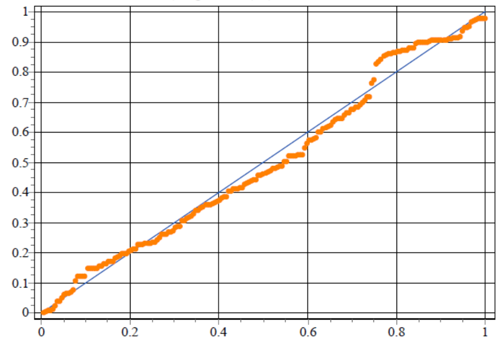

P (Empirical)

(L)

RBC | Lognormal (3P)

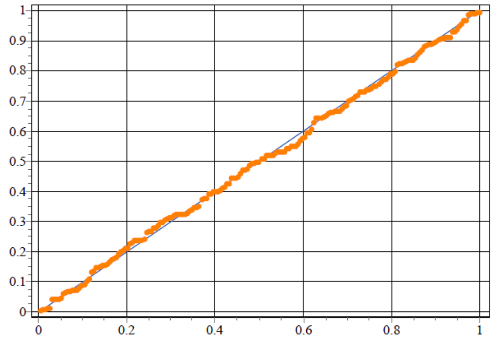

P (Empirical)

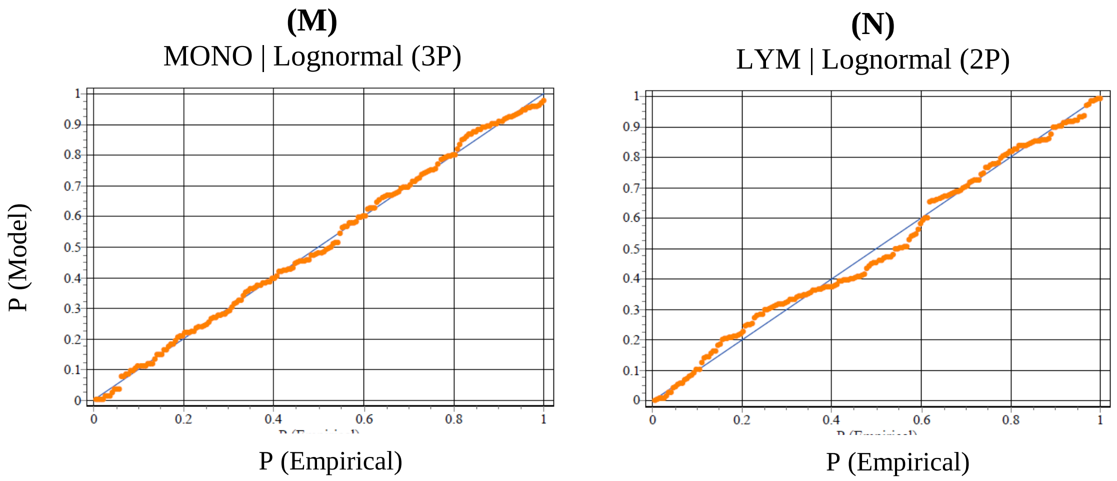

Figure S8: P-P plots for best-fitted distributions of features for ‘combination’ class

Table S4: Parameters of probability distributions followed by CBC features for ‘combination’ class

Features	Distribution	Parameters
HB	Burr (4P)	**k** = 310.15 | α = 5.0843 | β = 25.021 | γ = 1.7104
NEUT		**k** = 0.6936 | α = 0.8677 | β = 2.7632 | γ = 0.02
MCV	Burr (3P)	**k** = 1.1399 | α = 17.027 | β = 86.331
MCH		**k** = 1.5785 | α = 14.974 | β = 30.429
MCHC		**k** = 2.1927 | α = 22.674 | β = 35.626
BASO		**k** = 0.7544 | α = 0.9349 | β = 0.0937
HCT	Weibull (2P)	α = 5.6896 | β = 29.391
PLT		α = 0.9886 | β = 155.07
LYM%		α = 1.2255 | β = 37.432
MONO%	Gamma (3P)	α = 1.0501 | β = 14.484 | γ = 0.0954
WBC	Lognormal (3P)	σ = 1.9270 | μ = 2.3948 | γ = 0.2532
RBC		σ = 0.0872 | μ = 2.1107 | γ = -5.091
MONO		σ = 2.0404 | μ = 0.3205 | γ = 0.0038
LYM	Lognormal (2P)	σ = 1.5866 | μ = 1.0567

The above-mentioned parameters of the best-fitted probability distributions has been used to generate 500 random numbers for each class, resulting in 2000 synthetic instances that mimic the distributional properties of the original 287 instances. The original 287 and the synthetic 2000 instances are combined to generate a ‘hybrid’ synthetic dataset, which has been used in the downstream analysis.

REFERENCES

[1] A. Gonzales, G. Guruswamy, and S. R. Smith, "Synthetic data in health care: a narrative review," *PLOS Digital Health,* vol. 2, no. 1, p. e0000082, 2023.

[2] D. Rankin, M. Black, R. Bond, J. Wallace, M. Mulvenna, and G. Epelde, "Reliability of supervised machine learning using synthetic data in health care: Model to preserve privacy for data sharing," *JMIR medical informatics,* vol. 8, no. 7, p. e18910, 2020.

[3] K. Schittkowski, "EASY-FIT: a software system for data fitting in dynamical systems," *Structural and Multidisciplinary Optimization,* vol. 23, pp. 153-169, 2002.

[4] R. P. Shrestha *et al.*, "Models for the red blood cell lifespan," *Journal of pharmacokinetics and pharmacodynamics,* vol. 43, pp. 259-274, 2016.

[5] C. S. Sodhi, L. C. d. S. M. Ozelim, and P. N. Rathie, "Dielectric relaxation model of human blood as a superposition of Debye functions with relaxation times following a Modified-Weibull distribution," *Heliyon,* vol. 7, no. 3, 2021.

[6] P. R. Tadikamalla, "A look at the Burr and related distributions," *International Statistical Review/Revue Internationale de Statistique,* pp. 337-344, 1980.

[7] J. E. Mittler, B. Sulzer, A. U. Neumann, and A. S. Perelson, "Influence of delayed viral production on viral dynamics in HIV-1 infected patients," *Mathematical biosciences,* vol. 152, no. 2, pp. 143-163, 1998.

[8] E. L. Crow and K. Shimizu, *Lognormal distributions*. Marcel Dekker New York, 1987.

[9] K. C. Ayienda, *Gamma and related distributions*. BoD–Books on Demand, 2014.

[10] C. Lai, D. Murthy, and M. Xie, "Weibull distributions," *Wiley Interdisciplinary Reviews: Computational Statistics,* vol. 3, no. 3, pp. 282-287, 2011.

[11] G. Yari and Z. Tondpour, "The new Burr distribution and its application," *Mathematical Sciences,* vol. 11, no. 1, pp. 47-54, 2017.

[12] A. Ghasemi and S. Zahediasl, "Normality tests for statistical analysis: a guide for non-statisticians," *International journal of endocrinology and metabolism,* vol. 10, no. 2, p. 486, 2012.

[13] E. B. Holmgren, "The PP plot as a method for comparing treatment effects," *Journal of the American Statistical Association,* vol. 90, no. 429, pp. 360-365, 1995.
